# Tau fibrils induce glial inflammation and neuropathology via TLR2 in Alzheimer’s disease–related mouse models

**DOI:** 10.1172/JCI161987

**Published:** 2023-09-15

**Authors:** Debashis Dutta, Malabendu Jana, Ramesh Kumar Paidi, Moumita Majumder, Sumita Raha, Sridevi Dasarathy, Kalipada Pahan

**Affiliations:** 1 Department of Neurological Sciences, Rush University Medical Center, Chicago, Illinois, USA.; 2 Division of Research and Development, Jesse Brown Veterans Affairs Medical Center, Chicago, Illinois, USA.

**Keywords:** Inflammation, Neuroscience, Alzheimer disease, Cytokines

## Abstract

Glial activation and inflammation coincide with neurofibrillary tangle (NFT) formation in neurons. However, the mechanism behind the interaction between tau fibrils and glia is poorly understood. Here, we found that tau preformed fibrils (PFFs) caused induction of inflammation in microglia by specifically activating the TLR2/MyD88, but not the TLR4/MyD88, pathway. Accordingly, the WT TLR2–interacting domain of MyD88 (wtTIDM) peptide inhibited tau PFF–induced activation of the TLR2/MyD88/NF-κB pathway, resulting in reduced inflammation. Nasal administration of wtTIDM in P301S tau–expressing *PS19* mice was found to inhibit gliosis and inflammatory markers, as well as to reduce pathogenic tau in the hippocampus, resulting in improved cognitive behavior in *PS19* mice. The inhibitory effect of wtTIDM on tau pathology was absent in *PS19* mice lacking *TLR2*, reinforcing the essential involvement of TLR2 in wtTIDM-mediated effects in vivo. Studying the mechanism further, we found that the *tau* promoter harbored a potential NF-κB–binding site and that proinflammatory molecules increased transcription of *tau* in neurons via NF-κB. These results suggest that tau-induced neuroinflammation and neuropathology require TLR2 and that neuroinflammation directly upregulates tau in neurons via NF-κB, highlighting a direct connection between inflammation and tauopathy.

## Graphical abstract

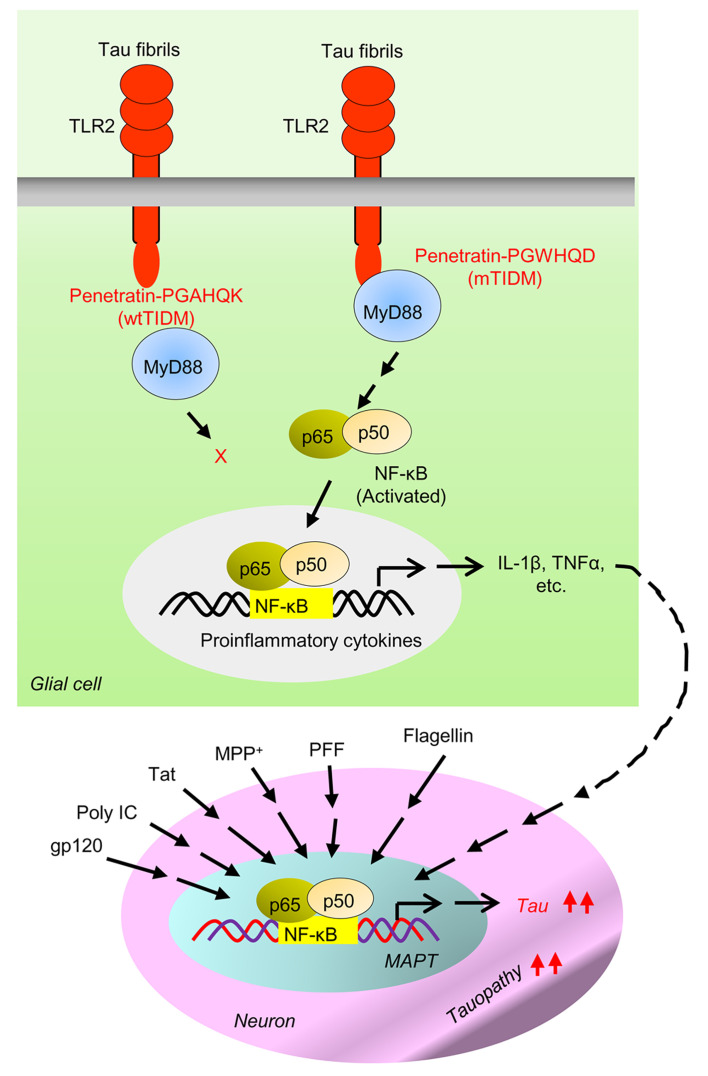

## Introduction

Tauopathy is defined as a progressive neurodegenerative disorder caused by the abnormal accumulation of the protein tau. The characteristic tau accumulation and formation of neurofibrillary tangles (NFTs) in multiple brain regions are well manifested in diseases including Alzheimer’s disease (AD), progressive supranuclear palsy (PSP), frontotemporal dementia (FTD), Pick’s disease, and corticobasal degeneration (CBD) (1, 2). Tau proteins in humans exist in 6 different isoforms resulting from the alternative splicing of the pre-mRNA forming 3R and 4R tau species (3). In AD, both the 3R and 4R isoforms are found, but specifically in neurons. In addition to their prevalent deposition in neurons, tau aggregates are also found in astrocytes, specifically in brains affected with PSP and CB, where isoforms of 4R tau are found in the filaments (4). The findings indicate that specific tau species found in the tangles differ among these diseases, and the mode of pathology may depend upon the brain region. In the case of AD-related tauopathy models, the existing literature indicates that tau inclusions emerge from the entorhinal cortex and travel through neuronal connections toward the hippocampus. Similarly, multiple studies using experimental animal models of AD pathology have demonstrated spreading of tau from the entorhinal cortex to the hippocampus (5, 6).

Microglia are the major brain-resident scavenger cells that actively take part in clearing pathogens, dying neurons, synapses, and aggregated proteins (7, 8). These physiological functions of microglia are indispensable for maintaining homeostasis in the developing and adult brain. However, according to Asai et al. (9), depletion of microglia halts tau propagation. Accordingly, exaggerated microglial inflammation has been well documented in preclinical animal models of tauopathy and convincingly demonstrated in human AD brains (10–14). Microglial activation is also found to coincide with the formation of phospho-tau aggregates in the hippocampus of neuron-specific tau–expressing *P301S* (*PS19*) mice (12, 15). However, the mechanism by which aggregated tau leads to microglial activation is poorly understood.

Here, we demonstrated that tau fibrils induced the activation of TLR2, but not TLR4, in microglial cells and that tau fibrils stimulated microglial inflammation via TLR2. Similarly, the WT TLR2–interacting domain of MyD88 (wtTIDM) peptide, capable of blocking the interaction of TLR2 with MyD88 (16), prevented tau-mediated TLR2 activation in microglia. Intranasal administration of wtTIDM in aged *PS19* mice resulted in significant inhibition of neuroinflammation concomitant with reduced NFT formation in neurons and an improvement in cognitive behavior. Genetic deletion of *TLR2* in *PS19* mice also halted tau pathology in the hippocampus of *PS19* mice. Most interestingly, we report that the tau promoter contained a consensus NF-κB–binding site, and therefore inflammatory molecules upregulated neuronal tau expression via NF-κB activation.

## Results

### Activation of microglia by fibrillar tau.

**Figure 1:**
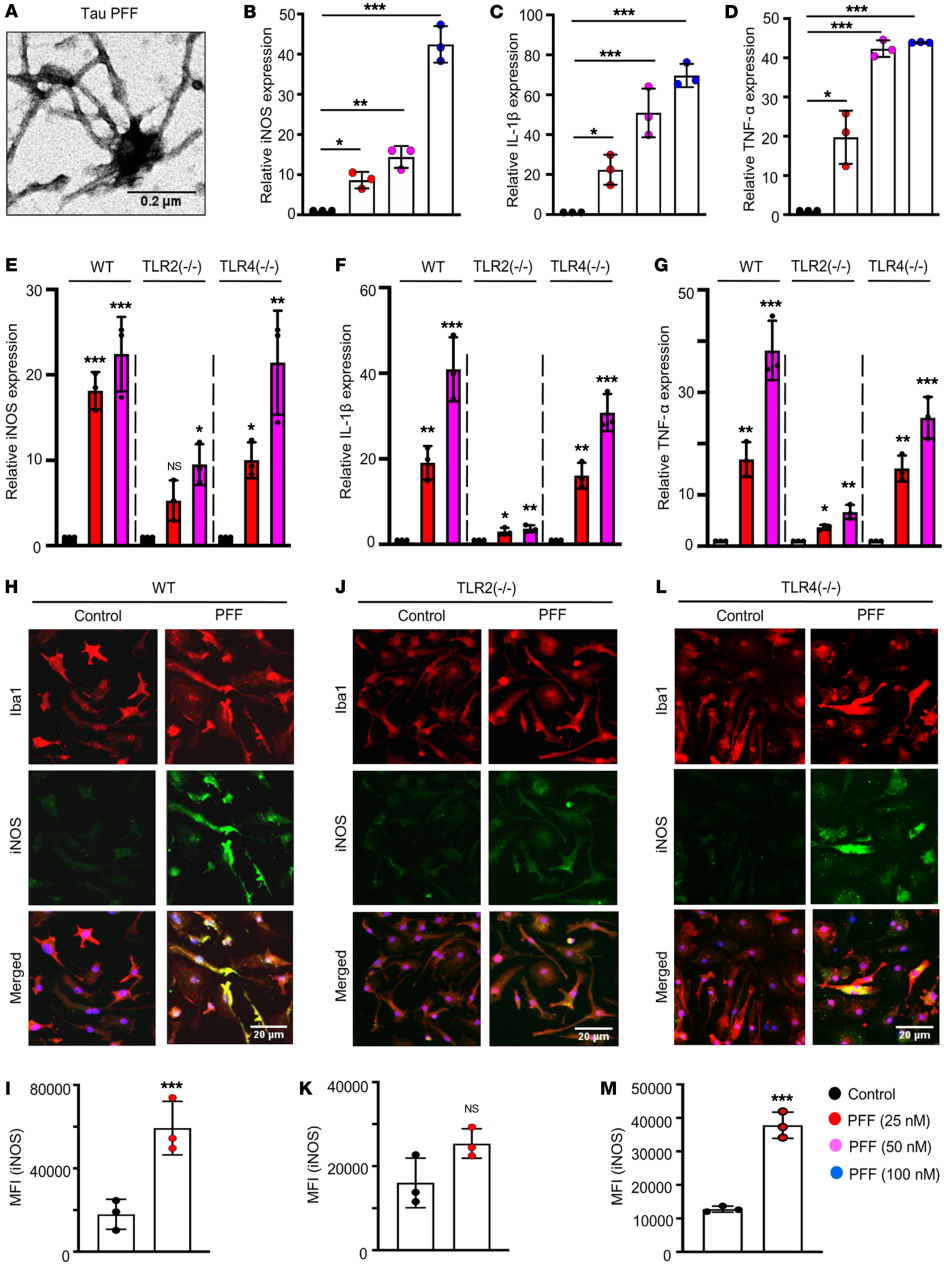
Tau PFFs induce microglial inflammation via TLR2. (**A**) Tau fibrils were prepared in vitro from full-length human tau monomers (2N4R isoform) and characterized by EM. Scale bar: 0.2 μm. (**B**–**D**) Induction of inflammatory molecules including iNOS, IL-1β, and TNF-α in WT primary microglia after treatment with different doses of tau PFFs (25, 50, and 100 nM) was measured by real-time PCR. Expression of iNOS (**E**), IL-1β (**F**), and TNF-α (**G**) in PFF-treated primary microglia derived from WT, *TLR2^–/–^*, and *TLR4^–/–^* pups was measured by real-time PCR. Protein expression of iNOS in PFF-induced primary microglia derived from WT (**H** and **I**), *TLR2^–/–^* (**J** and **K**), and *TLR4^–/–^* (**L** and **M**) pups was assessed by coimmunostaining of iNOS and Iba1, followed by MFI analysis of iNOS (green) using ImageJ. Scale bars: 20 μm. Statistical analyses among multiple groups were conducted using 1-way ANOVA followed by Tukey’s multiple-comparison analysis, whereas unpaired 2-tailed *t* test was conducted for comparing 2 groups. *P < 0.05, **P < 0.01, and ***P < 0.001 compared with the untreated control group. Values are presented as mean ± SD (*n* = 3 different experiments).

Human full-length tau (2N4R) monomers were subjected to in vitro fibrillation in the presence of heparin for 7 days at 37°C, and then this solution was centrifuged at 100,000*g* to precipitate the tau fibrils. This protein preparation was imaged under an electron microscope to validate successful generation of tau preformed fibrils (PFFs) (Figure 1A). By using the ToxinSensor Chromogenic LAL Endotoxin Assay Kit (GenScript), we also did not find any endotoxin contamination in PFFs (standard curve, Supplemental Figure 1A; level of LPS, Supplemental Figure 1B; supplemental material available online with this article; https://doi.org/10.1172/JCI161987DS1). Then primary WT microglia were challenged with different concentrations of tau PFFs for 5 hours, followed by monitoring of the mRNA expression of several inflammatory molecules, such as inducible NOS (iNOS), IL-1β, and TNF-α by real-time PCR (Figure 1, B–D). The results demonstrated a dose-dependent increase in expression of these inflammatory molecules, and even the lowest dose of PFFs (25 nM) caused a 10-fold increase in iNOS expression and around 20-fold upregulation of both IL-1β and TNF-α compared with the untreated control cells. The results indicated that tau PFFs, at a very low dose, were capable of inducing substantial inflammation in microglia.

### Tau PFFs activate microglia via TLR2, not TLR4.

Induction of inflammatory genes in microglia is a result of signaling cascades that initiate from the binding of extracellular stimulus with specific membrane receptor proteins. Of the numerous receptors involved in activating inflammatory pathways in microglia, TLRs have received significant attention as potential receptors of aggregated proteins, including amyloid β (Aβ) and α-synuclein (17–19). Therefore, to test the hypothesis that tau PFF–induced activation of microglia is TLR dependent, we performed gene expression analyses of inflammatory markers in WT and *TLR2^–/–^* microglia. As described above, expression of *iNOS*, *IL-1β*, and *TNF-α* was upregulated by several folds in PFF-induced WT microglia (Figure 1, E–G). Surprisingly, this substantial increase in expression of inflammatory genes was not found in PFF-treated *TLR2^–/–^* microglia (Figure 1F). Although subtle upregulation of these genes was observed in *TLR2^–/–^* microglia, the level of fold change compared with the control group was much lower in these microglia than in WT microglia, indicating that tau PFFs might require TLR2 to activate microglia. To further validate the results, we performed immunocytochemistry for iNOS in both WT and *TLR2^–/–^* microglia; significant increases in iNOS levels were observed following PFF treatment only in WT cells (Figure 1, H and I), whereas there was no significant upregulation in *TLR2^–/–^* cells (Figure 1, J and K). However, significant induction of *iNOS*, *IL-1β*, and *TNF*-*α* genes was found in *TLR4^–/–^* microglia following PFF treatment, and the fold change in gene expression was comparable to that in WT microglia (Figure 1, E–G). PFF-induced upregulation of iNOS protein was also confirmed in *TLR4^–/–^* microglia (Figure 1, L and M). Furthermore, ELISA of supernatants showed that PFFs induced increased production of TNF-α (Supplemental Figure 2A) and IL-1β (Supplemental Figure 2B) proteins in microglia isolated from WT and *TLR4^–/–^*, but not *TLR2^–/–^*, mice. These results suggest that tau PFFs required TLR2, but not TLR4, to induce the activation of microglia.

Along with microglia, activation and resulting inflammation of astrocytes are also reported in tauopathy brains (20, 21). Therefore, we carried out similar experiments in mouse primary astrocytes. The results demonstrated that tau PFFs induced mRNA expression of IL-1β, TNF-α, and iNOS in both WT and *TLR4^–/–^* astrocytes, but not in *TLR2^–/–^*, astrocytes (Supplemental Figure 3, A–C). Accordingly, WT, *TLR2^–/–^*, and *TLR4^–/–^* astrocytes were transfected with luciferase plasmid constructs containing promoters of IL-1β, TNF-α, and iNOS and then challenged with tau PFFs. The results showed significant increases in luciferase activity following PFF exposure in WT and TLR4^–/–^ astrocytes, but not in TLR2^–/–^ astrocytes (Supplemental Figure 3, D–F). Activation of inflammation was also validated by immunocytochemistry of iNOS in glial fibrillary acidic protein–positive (GFAP^+^) primary astrocytes, where upregulation of iNOS expression by tau PFF stimulation was confirmed in WT and *TLR4^–/–^* astrocytes, whereas this effect was absent in *TLR2^–/–^* astrocytes (Supplemental Figure 3, G and H).

### AD brain–derived tau activates microglia via TLR2.

Since synthetic tau PFFs required TLR2 to induce the activation of microglia, we examined whether AD brain–derived tau (AD-Tau) also needed TLR2 for microglial activation. As evident from double-label immunofluorescence of Iba1 and iNOS (Supplemental Figure 4A), different doses of AD-Tau clearly caused the activation of WT microglia. This was confirmed by MFI quantification of Iba1 (Supplemental Figure 4C) and iNOS (Supplemental Figure 4D). Real-time analysis also indicated upregulation of *iNOS* (Supplemental Figure 4E), *TNF-α* (Supplemental Figure 4F), *IL-1β* (Supplemental Figure 4G), and *CD11b* (Supplemental Figure 4H) mRNAs in WT microglia by AD-Tau. However, similar to synthetic tau PFFs, AD-Tau at different doses remained unable to activate microglia isolated from *TLR2^–/–^* mice as compared with WT microglia (Supplemental Figure 4, A–D). AD-Tau also could not upregulate the mRNA expression of *iNOS* (Supplemental Figure 4E), *TNF-α* (Supplemental Figure 4F), *IL-1β* (Supplemental Figure 4G), and *CD11b* (Supplemental Figure 4H) mRNAs in *TLR2^–/–^* microglia.

### Tau PFFs induce the activation of TLR2, not TLR4.

**Figure 2:**
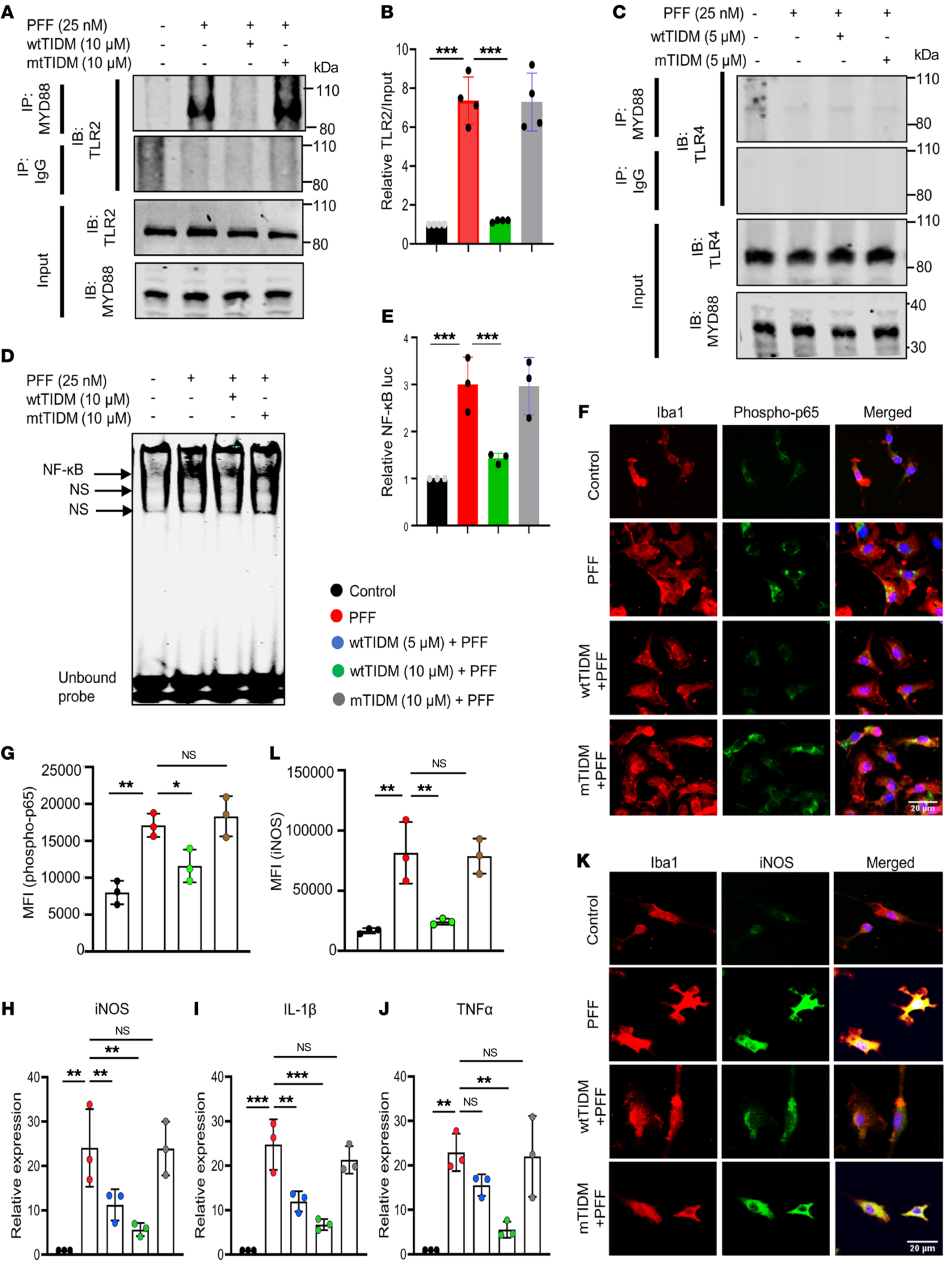
The wtTIDM peptide inhibits tau PFF–induced TLR2-MyD88 interaction and NF-κB activation in microglia. (**A**) BV2 cells were pretreated with wtTIDM or mTIDM (10 μM), followed by exposure to PFFs (25 nM), and after 1 hour of PFF administration, the TLR2-MyD88 interaction was monitored by IP. Input samples were probed for both anti-TLR2 and anti-MyD88 antibodies. (**B**) Densitometry shows the MyD88-bound TLR2 level compared with the input. (**C**) The interaction of TLR4 with MyD88 was also assessed in BV2 cells by IP under identical experimental conditions. NF-κB activation was measured in nuclear extracts isolated from TIDM-treated, PFF-exposed BV2 cells by EMSA (**D**) and by luciferase assay, wherein cells were initially transfected with luciferase reporter gene constructs (**E**). (**F** and **G**) The level of activated NF-κB in TIDM-treated, PFF-exposed primary mouse microglia was assessed by immunostaining of the phospho-Ser536 form of p65 in Iba1^+^ microglia, followed by MFI analysis of phospho-p65. (**H**–**J**) Primary microglia were pretreated with wtTIDM or mTIDM (5 and 10 μM) and then challenged with tau PFFs for 5 hours, followed by expression analysis of inflammatory genes (iNOS, IL-1β, and TNF-α) by real-time PCR. (**K**) The level of iNOS protein expression in TIDM-treated primary microglia was analyzed after 16 hours of PFF exposure by immunostaining. Scale bars: 20 μm. (**L**) MFI of iNOS expression was measured by ImageJ. Statistical analyses were performed by 1-way ANOVA, followed by Tukey’s multiple-comparison analysis. *P < 0.05, **P < 0.01 and ***P < 0.001 compared with the designated groups. Values are presented as mean ± SD (*n* = 3 different experiments).

Induced activation of membrane-bound TLR2 by its agonist increases its interaction with the adapter protein MyD88 at the cytosolic part of the cell (16). To monitor the effect of tau PFFs on TLR2-MyD88 interaction, we employed immunoprecipitation (IP) coupled with Western blot analyses. In this case, BV2 microglial cells were treated with tau PFFs (25 nM) for 1 hour, and the membrane fractions of experimental cells were processed for IP analysis, which showed increases in binding of TLR2 with MyD88 following PFF treatment (Figure 2, A and B). In contrast, tau PFFs remained unable to stimulate the interaction between TLR4 and MyD88, indicating the specificity of the effect and suggesting no involvement of activated TLR4 following PFF exposure (Figure 2C). Since activation of the TLR2/MyD88 pathway is known to transduce the activation of the proinflammatory transcription factor NF-κB, which is essential for transcription of different proinflammatory molecules, we monitored NF-κB activation. While the DNA-binding activity of NF-κB was evaluated by the formation of a distinct and specific complex in a gel shift DNA binding assay, the transcriptional activity of NF-κB was monitored by the expression of luciferase from a reporter construct, *pNF-κB-Luc* (16, 22). A substantial increase in DNA binding (Figure 2D) and significant increase in transcriptional activity (Figure 2E) of NF-κB was found in PFF-exposed microglial cells, confirming the induction of NF-κB activation by tau PFFs in microglia.

### Tau PFF–induced TLR2 activation is inhibited by wtTIDM peptide.

The wtTIDM peptide was characterized in our previous study, where we demonstrated the efficacy of this peptide in specifically blocking the interaction between TLR2 and MyD88 (16). In the present study, TIDM peptides were used to further validate the specificity of tau PFF–mediated activation of the TLR2/MyD88 pathway. In this case, wtTIDM or mutated TIDM (mTIDM) peptides were added to cells prior to PFF exposure. IP analysis revealed increased interaction of TLR2 with MyD88 only in PFF-treated microglia as compared with untreated cells (Figure 2, A and B). However, markedly less TLR2-MyD88 interaction was observed in cells receiving wtTIDM, but not mTIDM (Figure 2, A and B). Furthermore, findings of EMSA and luciferase assay showed reduced DNA binding and transcriptional activity of NF-κB in wtTIDM-treated, but not mTIDM-treated, PFF-exposed microglia (Figure 2, D and E). This result not only confirms that tau PFFs induce TLR2-MyD88 interaction, but also highlights the fact that wtTIDM can be used as a molecule to inhibit PFF-induced microglial activation. This finding was corroborated in WT microglia by monitoring the level of the phospho-Ser536 activated form of p65, the subunit of NF-κB. A significantly higher level of phospho-p65 was found in PFF-treated primary microglia, whereas the level was lower in wtTIDM-treated, but not in mTIDM-treated, cells (Figure 2, F and G). To evaluate the inflammatory response in wtTIDM-treated cells, we carried out gene expression analyses of inflammatory molecules. The data exhibited drastic downregulation of iNOS, IL-1β, and TNF-α by wtTIDM, but not mTIDM (Figure 2, H–J). Last, the reduction in iNOS protein level in wtTIDM-treated microglia validated its antiinflammatory effect against tau PFFs (Figure 2, K and L). Together, these findings ensure that tau PFF–induced microglial inflammation requires TLR2 activation, and therefore, the potent TLR2 inhibitor wtTIDM is of prime importance to inhibit this effect.

### AD-Tau–induced microglial activation is also inhibited by wtTIDM peptide.

Since the wtTIDM peptide suppressed tau PFF–induced microglial activation, we examined whether AD-Tau–mediated microglial activation was also sensitive to wtTIDM peptide. As described above, AD-Tau strongly upregulated the mRNA expression of TNF-α (Supplemental Figure 5A), IL-1β (Supplemental Figure 5B), and iNOS (Supplemental Figure 5C) in WT microglia. Consistent with the results seen with synthetic tau PFFs, AD-Tau–induced mRNA expression of proinflammatory molecules was also inhibited by wtTIDM, but not mTIDM, peptide (Supplemental Figure 5, A–C). As evident from double-label immunofluorescence of Iba1 and iNOS (Supplemental Figure 5D), wtTIDM, but not mTIDM, peptide strongly suppressed the level of IbaI (Supplemental Figure 5E) and iNOS (Supplemental Figure 5F) proteins in AD-Tau–stimulated WT microglia.

### The wtTIDM peptide inhibits glial inflammation in tauopathy.

The inhibitory effect of wtTIDM against synthetic tau PFF– and AD-Tau–induced microglial inflammation led us to investigate its effect in an in vivo model of tauopathy. *PS19* mice expressing P301S mutated human tau protein specifically in neurons are known to develop tau pathology with characteristic NFT formation by 6 months of age, whereas neuronal degeneration in the hippocampus is observed by 9 months of age (15). Interestingly, glial activation precedes formation of NFTs in the hippocampus. Given the fact these mice exhibit glial activation and inflammation prior to neuronal death, we first evaluated the status of TLR2 and MyD88 proteins in the resident microglia of hippocampus in *PS19* mice 2 months ahead of the start of neurodegeneration (at 7 months of age). We found higher expression of TLR2 as well as MyD88 in Iba1^+^ microglia in these mice than age matched nontransgenic (*nTg*) mice (Supplemental Figure 6, A–D). The endogenous upregulation of TLR2 and MyD88 was also recapitulated in hippocampal astrocytes of *PS19* mouse brain (Supplemental Figure 7, A–D). More importantly, enhanced expression of TLR2 and MyD88 coincided with aggravated microgliosis (Supplemental Figure 6E) as well as astrogliosis (Supplemental Figure 7E). Although this finding does not make it clear whether TLR2 and MyD88 upregulation was a result of initial tau pathology in *PS19* mice, it does reveal that their upregulation paralleled glial activation in the hippocampus. Therefore, next we examined the role of TLR2/MyD88 in gliosis and inflammation. To achieve that purpose, we started wtTIDM/mTIDM nasal administration (0.1 mg/kg) in *PS19* mice at the age of 7 months. After 1.5 months of TIDM treatment, multiple biochemical analyses were performed on samples from the hippocampal tissues.

Recently, we demonstrated that after intranasal administration, wtTIDM peptide is capable of entering the hippocampus (16). Here, we examined whether after intranasal administration, the wtTIDM peptide was capable of reducing the association between TLR2 and MyD88 in vivo in the hippocampus of *PS19* mice. As evident from triple-labeling of hippocampal sections with Iba1, TLR2, and MyD88, followed by imaging under super-resolution (Airyscan, Zeiss) confocal microscopy, TLR2 and MyD88 colocalized in Iba1^+^ microglia in the hippocampus of control untreated *PS19* mice (Supplemental Figure 8, A and B). However, this association between TLR2 and MyD88 in Iba1^+^ microglia decreased in the hippocampus of *PS19* mice treated with intranasal wtTIDM, but not mTIDM, peptide (Supplemental Figure 8, A and B).

**Figure 3:**
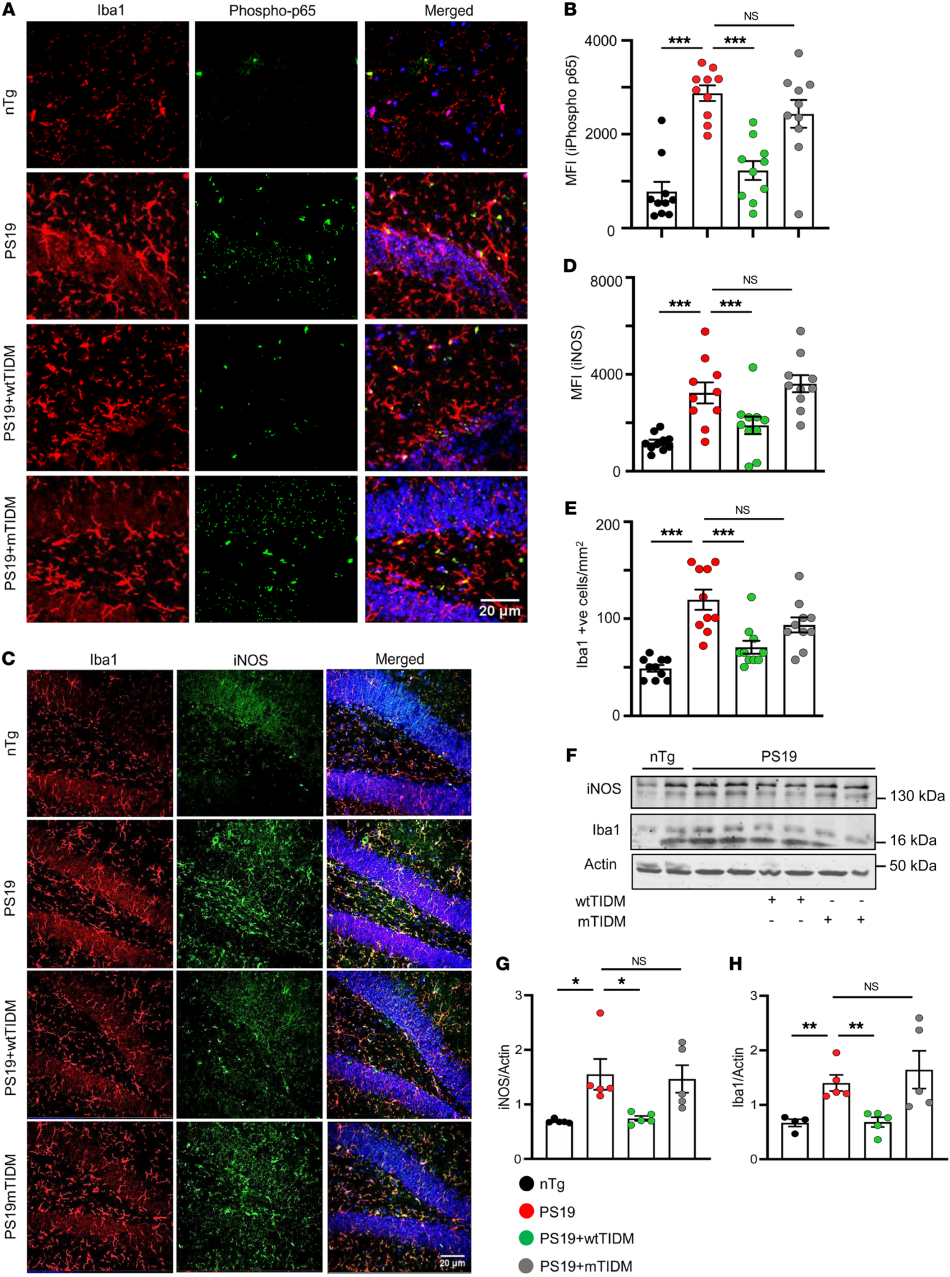
The wtTIDM nasal administration suppresses gliosis and inflammation in the hippocampus of *PS19* animals. (**A**) *PS19* mice (7 months old) were given intranasal administration of wtTIDM or mTIDM (0.1 mg/kg) for 1.5 months, and then activation of NF-κB in resident microglia of hippocampus was monitored by double-label immunofluorescence analysis of phospho-p65 in Iba1^+^ cells. (**B**) Images were captured at 20× magnification and zoomed to visualize phospho-p65 localization. Expression of phospho-p65 (green) was measured by MFI analysis. (**C**) Similarly, expression of iNOS in hippocampal microglia was monitored by double immunofluorescence analysis of iNOS and Iba1. Images were captured at 20× magnification. Scale bars: 20 μm. (**D**) iNOS expression (green) in Iba1^+^ cells was measured using ImageJ. (**E**) The number of Iba1^+^ cells in both the CA1 and DG regions was determined by the manual counting option provided in ImageJ and expressed as cells per mm^2^ of area. (**F**) For both MFI and counting analyses, microglia present in both CA1 and DG were considered. Two sections from each brain were included for immunofluorescence analysis, and the value obtained from each section is represented in the bar diagram. The protein level of iNOS as well as Iba1 in hippocampal tissue was also measured by Western blotting, and actin was used the loading control. (**G** and **H**) Band densities of iNOS and Iba1 were presented with respect to that of actin. Statistical analyses were performed following 1-way ANOVA followed by Tukey’s multiple-comparison analysis. *P < 0.05, **P < 0.01, and ***P < 0.001 compared with the designated groups. Values are presented as mean ± SEM (*n* = 5 different animals).

Next we examined the effect of TIDM peptides on microglial activation in vivo in the hippocampus. Similar to that in the PFF-exposed primary microglia, the level of the activated RelA subunit of NF-κB (phospho-Ser536-p65) was higher in hippocampal microglia of *PS19* brains as compared with *nTg* brains (Figure 3, A and B). Accordingly, expression of the downstream inflammatory protein iNOS in microglia was also higher in *PS19* than in *nTg* mice (Figure 3, C and D). The induction of inflammation accompanied by an increased number of Iba1^+^ microglia signifies the occurrence of microgliosis (Figure 3E). However, treatment of *PS19* mice with wtTIDM, but not mTIDM, markedly inhibited the activation of p65, upregulation of iNOS, and overall microgliosis in the hippocampus (Figure 3, A–E). Furthermore, Western blot analyses confirmed the specific inhibitory effect of wtTIDM on microgliosis and inflammation, as reduced levels of Iba1 and iNOS were found in wtTIDM-treated *PS19* mice as compared with untreated *PS19* mice (Figure 3, F–H). We also examined astroglial activation and found that iNOS expression and the number of GFAP^+^ cells were higher in astrocytes of *PS19* brains as compared with *nTg* brains (Supplemental Figure 9, A–C). However, similar to the suppression of microglial inflammation, treatment of *PS19* mice with wtTIDM, but not mTIDM, led to a reduction in iNOS in astrocytes (Supplemental Figure 9, A–C). Collectively, these data establish the fact that inhibition of TLR2 activation by wtTIDM reduced gliosis and inflammation in the hippocampus of *PS19* mice.

### wtTIDM peptide–mediated TLR2 inhibition mitigates NFT formation in neurons.

**Figure 4:**
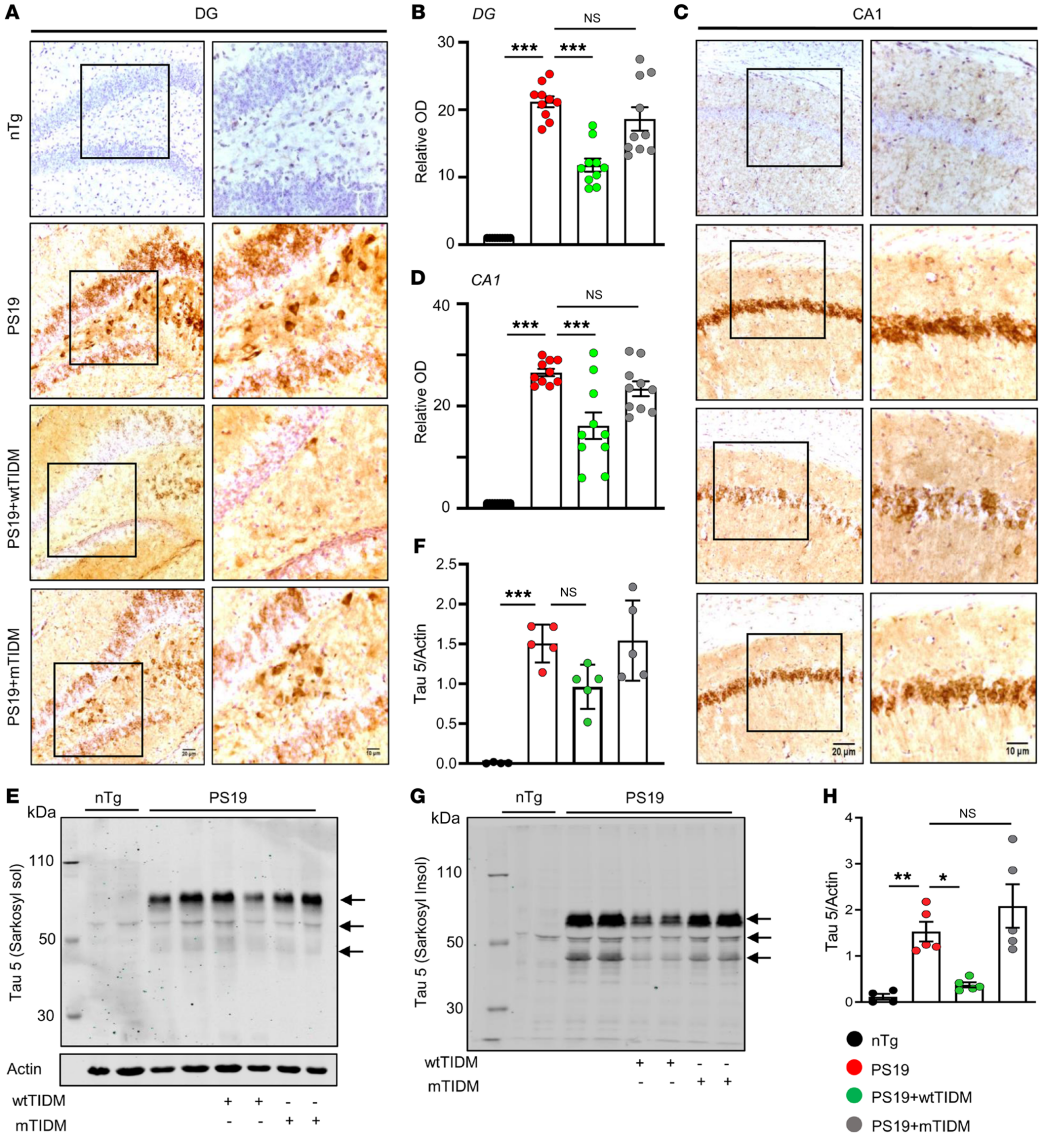
wtTIDM nasal administration alleviates aggregated tau deposition in the hippocampus of *PS19* animals. *PS19* mice (7 months old) were given intranasal administration of wtTIDM or mTIDM (0.1 mg/kg) for 1.5 months, and content of NFTs in hippocampal neurons was assessed by immunohistochemistry using antibody specific for total tau (Tau-5). Images obtained from the DG (**A**) and CA1 (**C**) brain regions of the experimental *PS19* mice are shown at 20× and 40× magnifications. Scale bars: 20 μm (left columns), 10 μm (right columns). (**B** and **D**) Relative OD of Tau-5 staining compared with the *nTg* mice was measured using Fiji. Two sections from each brain were included for immunostaining analysis, and the value obtained from each section is represented in the bar diagram. The total level of tau present in sarkosyl-soluble (sol) (**E**) and sarkosyl-insoluble (Insol) (**G**) tissue fractions was assessed by Western blotting. (**F**) The expression of total tau in the sarkosyl-soluble fraction was represented with respect to the actin present in the sarkosyl-soluble fraction. (**H**) On the other hand, the level of total tau in the sarkosyl-insoluble fraction was represented with respect to the actin present in the sarkosyl-soluble fraction, as an actin band was not found in the sarkosyl-insoluble fraction. Arrows indicate the different isomers of tau, and the band near 70 kDa obtained from the sarkosyl-soluble fraction was considered for density analysis. Statistical analyses were conducted following 1-way ANOVA followed by Tukey’s multiple-comparison analysis. ***P < 0.001 compared with the respective groups. Values are presented as mean ± SEM (*n* = 5 different animals).

Inhibition of glial inflammation was previously shown to attenuate tau pathology and related neurodegeneration in *PS19* mice (15, 23). This prompted us to evaluate the effect of wtTIDM on NFT formation in neurons. Immunohistochemistry was performed for total tau protein using Tau-5 antibodies to monitor the level of tau deposition. The results demonstrated exaggerated NFT formation in granular cells of the DGs (Figure 4, A and B) and pyramidal neurons of CA1 (Figure 4, C and D) of *PS19* mouse brains. In contrast, wtTIDM treatment drastically alleviated aggregated tau pathology in these neurons. However, mTIDM treatment did not result in any reduction in NFT formation (Figure 4, A–D), indicating the specificity of the effect. Similarly, immunohistochemistry with PHF1 antibodies indicated deposition of phospho-Ser396/Ser404 tau in CA3 and DG of *PS19* mice that was reduced by intranasal treatment with wtTIDM, but not mTIDM, peptide (Supplemental Figure 10, A and B). Although we did not detect widespread phospho-Ser202-Thr205 tau in the hippocampus of 6-month-old *PS19* mice by AT8 staining, we found its accumulation when the mice were 8.5 months old (Supplemental Figure 11, A and B). However, alleviation of total tau deposition in wtTIDM-treated 8.5-month-old *PS19* mice was accompanied by a reduction in Ser202 and Thr205 phosphorylated tau accumulation, as revealed by immunostaining using AT8 antibodies (Supplemental Figure 12, A and B). Again, the mTIDM peptide had no such effect (Supplemental Figure 12, A and B), indicating specificity. To substantiate the data obtained from immunostaining, we conducted Western blotting on samples from hippocampal tissue fractions of experimental mice. No significant change in sarkosyl-soluble tau isomers (50–70 kDa) was found in either wtTIDM- or mTIDM-treated compared with untreated *PS19* brains (Figure 4, E and F). On the other hand, a reduced level of total tau was observed in the sarkosyl-insoluble fraction isolated from the wtTIDM-treated *PS19* brains in comparison to either untreated or mTIDM-treated *PS19* brains (Figure 4, G and H). This finding indicates that it was not the soluble tau, but the formation of pathological tau aggregate that was affected by intranasal wtTIDM administration.

### The wtTIDM peptide protects synaptic plasticity in PS19 mice.

**Figure 5:**
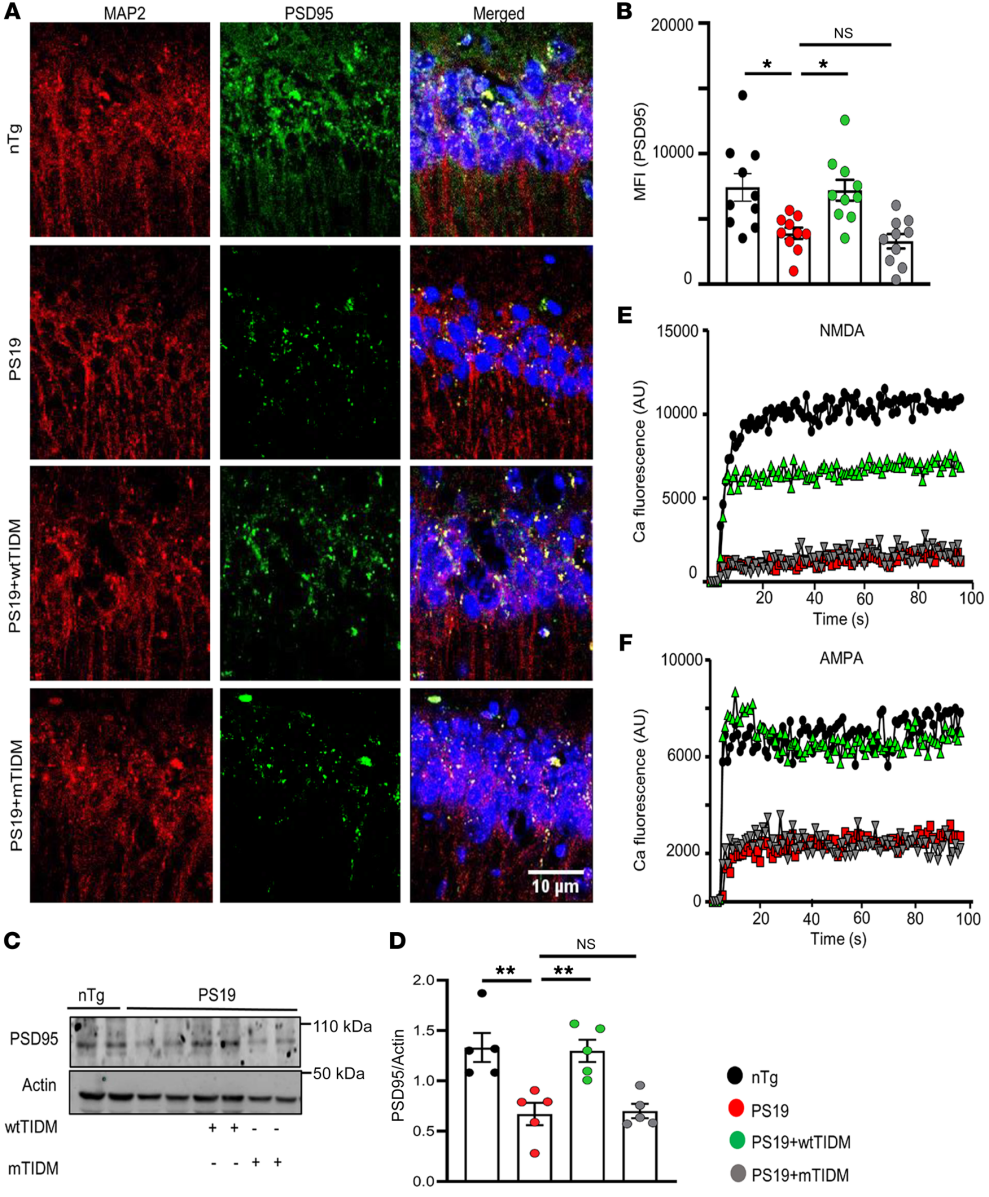
Hippocampal synaptic plasticity is retained by nasal wtTIDM treatment in *PS19* mouse brains. (**A**) *PS19* mice (7 months old) were given intranasal administration of wtTIDM or mTIDM (0.1 mg/kg) for 1.5 months. Hippocampal plasticity was primarily monitored by evaluating the level of PSD95 in pyramidal neurons of CA1 by double immunofluorescence of PSD95 and MAP2. Scale bar: 10 μm. (**B**) Expression of PSD95 (green) surrounding each DAPI^+^ nucleus in the pyramidal layer was measured by drawing the region of interest, then using the analyze-measure tool of ImageJ. The MFI data obtained from each section of a particular mouse brain are shown in the bar diagram. (**C** and **D**) Similarly, overall protein content of PSD95 in the hippocampus was assessed by Western blot analysis. *P < 0.05 and **P < 0.01 compared with the corresponding groups. Values are presented as mean ± SEM (*n* = 5 different animals). (**E**) NMDA-dependent Ca influx in hippocampal slices from experimental animals was measured by treating the slices with NMDA and NASPM (for blocking AMPA-mediated Ca influx). (**F**) Similarly, AMPA-dependent Ca influx was measured by using AMPA along with N20C (NMDA open-channel blocker). Fluorescence based Ca influx was monitored for 300 repeats in a PerkinElmer VICTOR X2 fluorimeter. The experiment was conducted on samples from 3 different mouse brains of each group.

Loss of synaptic function and reduction in synaptic proteins are some important pathological features found in tauopathy brains even prior to neuronal demise (24, 25). Postsynaptic density 95 (PSD95) is one of those vital postsynaptic scaffold proteins that have been shown to be downregulated in pyramidal neurons in the hippocampus under the burden of phosphorylated tau. Here, we also found loss of PSD95 in microtubule-associated protein 2–containing (MAP2-containing) neurons of CA1 (Figure 5, A and B) and CA3 (Supplemental Figure 13, A and B) of 8.5-month-old *PS19* mice. Furthermore, Western blot analysis confirmed overall loss of PSD95 in hippocampal tissues of these mice (Figure 5, C and D). However, loss of PSD95 was remarkably attenuated in CA1 (Figure 5, A and B) and CA3 (Supplemental Figure 13, A and B) of *PS19* mice treated with wtTIDM, but not mTIDM, peptide. Similarly, we also observed a loss of synaptophysin in CA3 of 8.5-month-old *PS19* mice that was significantly restored by intranasal administration of wtTIDM, but not mTIDM, peptide (Supplemental Figure 14, A and B). Next, the functional integrity of hippocampal neurons was measured by assessing Ca influx in hippocampal slices. Parallel to the loss of PSD95, reduced Ca influx through ionotropic glutamate receptors — including NMDA and α-amino-3-hydroxy-5-methyl-4-isoxazolepropionic acid (AMPA) — was observed in hippocampus of *PS19* mice as compared with age-matched *nTg* animals (Figure 5, E and F). However, Ca influx through NMDA and AMPA receptors was markedly improved in wtTIDM-treated *PS19* mice, and the value obtained for this group was almost the same as that in *nTg* mice (Figure 5, E and F). In contrast, an increase in PSD95 level or corrected Ca influx was not found in mTIDM-treated *PS19* animals. The overall finding is of importance as improved synaptic functioning, which is essential for memory formation by hippocampal neurons, was achieved by wtTIDM administration, and that happened with concomitant downregulation of NFT formation in *PS19* mouse brain.

### The wtTIDM peptide ameliorates cognitive deficits in PS19 animals.

**Figure 6:**
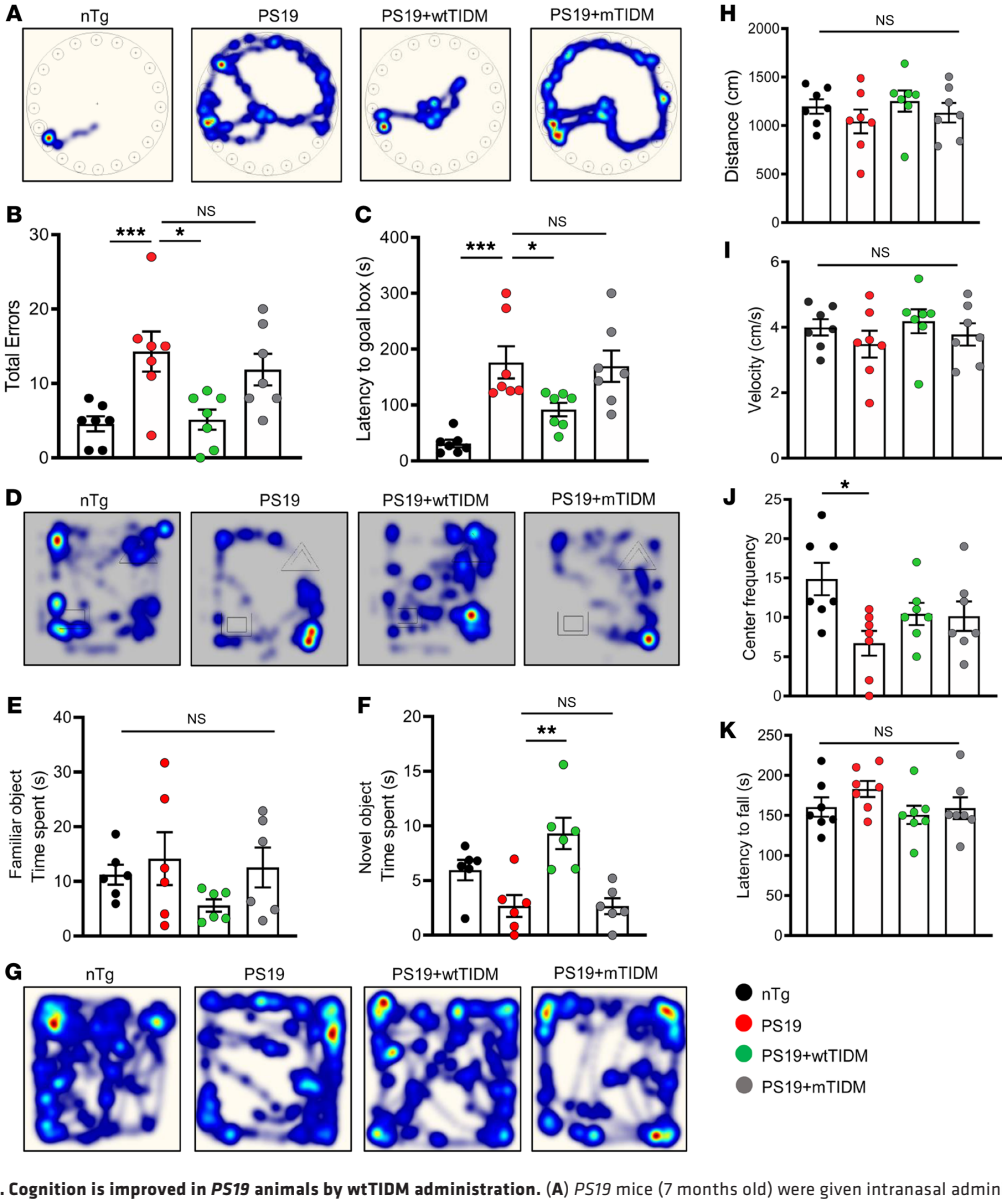
Cognition is improved in *PS19* animals by wtTIDM administration. (**A**) *PS19* mice (7 months old) were given intranasal administration of wtTIDM or mTIDM (0.1 mg/kg) for 1 month, and then spatial memory of these animals was evaluated by conducting Barnes maze analysis; the heatmaps demonstrate the exploratory activity of the animals in the maze to find out the goal box (*n* = 7 animals per group). Cognitive parameters including total errors made before reaching the goal box (**B**) and latency time for the reaching goal box (**C**) are shown in the diagrams. (**D**) NORT was conducted to explore the memory-retention ability of the experimental mice. Time spent by each mouse with the familiar object (**E**) and with the novel object.\ (**F**) was recorded for determining the cognitive performance of different groups of mice (*n* = 6 animals per group). Locomotor activity of mice was assessed by performing an open-field test (**G**), in which parameters including distance (**H**), velocity (**I**), and center frequency (**J**) in the arena were recorded. (**K**) Motor coordination was evaluated by rotarod test (*n* = 7 animals per group). Statistical analyses were performed with 1-way ANOVA followed by Tukey’s multiple-comparison analysis. *P < 0.05, **P < 0.01, and ***P < 0.001 compared with the designated groups. Values are presented as mean ± SEM.

We conducted cognitive tests to analyze the functional outcome of pathological tau reduction and improved synaptic function in the hippocampus by wtTIDM treatment. Barnes maze test exhibited impaired spatial learning and memory of untreated *PS19* animals compared with the *nTg* group, and this was evidenced by errors and latency (Figure 6, A–C). Similarly, in the novel object recognition test (NORT), *PS19* mice showed significantly lower preference for the novel object than did *nTg* mice (Figure 6, D–F). However, wtTIDM-treated *PS19* mice made fewer errors in the Barnes maze and thereby reached the goal box much earlier than the untreated *PS19* mice (Figure 6, A–C). In addition, wtTIDM-treated mice were found to have increased proclivity toward the novel object, and spent a longer time with it in the NORT (Figure 6, D–F). This specific behavioral improvement was not observed in the case of mTIDM-treated *PS19* animals. We also conducted motor behavioral tests in these mice, considering the fact that *PS19* mice start developing impaired motor activity as they age and eventually experience limb paralysis (15). However, in our experiments on *PS19* mice, we observed no drastic or significant changes in horizontal motor activity or maintaining motor coordination. The experimental mice, including *nTg*, untreated *PS19*, and TIDM-treated *PS19* mice, showed comparable distance moved (Figure 6, G and H), velocity (Figure 6I) in the arena, and time spent on the rotarod (Figure 6K). Only the time spent by *PS19* animals in the center of the arena was found to be significantly shorter than that by *nTg* mice; however, wtTIDM treatment was not found to have any effect on these parameters (Figure 6, G–J). Together, these data indicate restoration of learning capability and memory consolidation in mice with tauopathy after intranasal treatment with wtTIDM.

### The wtTIDM peptide–mediated decrease in NFT formation and improvement of cognitive functions were TLR2 dependent.

**Figure 7:**
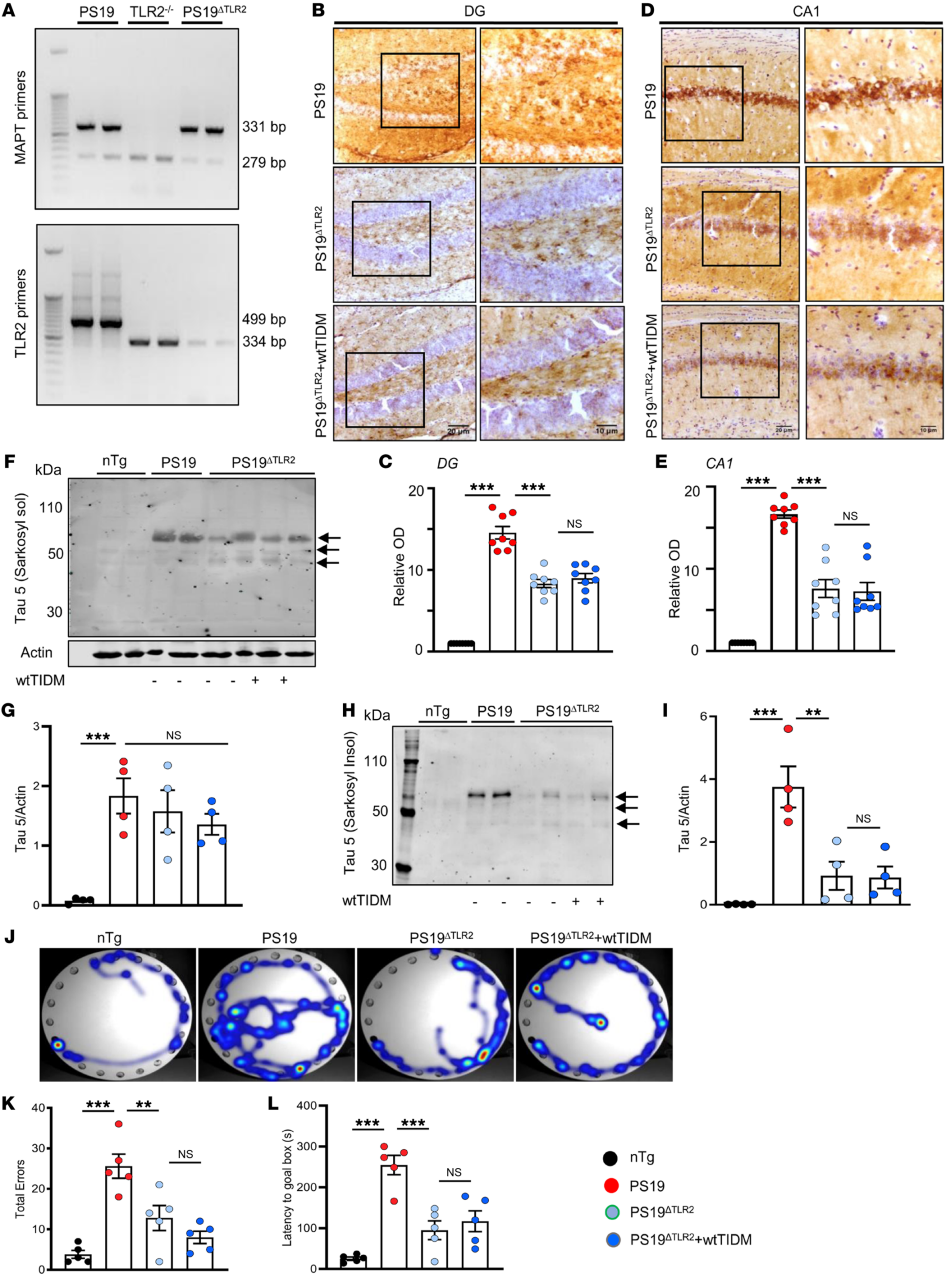
The wtTIDM treatment fails to reduce tau pathology and improve cognitive behavior in *PS19* mice lacking *TLR2*. (**A**) *PS19* mice were bred with *TLR2^–/–^* mice to obtain double-transgenic *PS19*^ΔTLR2^ mice. These mice were validated by genetic screening, where the 331 bp and 279 bp bands corresponded to *nTg* and *PS19* mice, respectively. Similarly, the 499 bp and 334 bp bands indicate *nTg* and *TLR2^–/–^* mice respectively. *PS19*^ΔTLR2^ mice (7 months old) received wtTIDM (0.1 mg/kg/d) nasal administration for 1 month; and at 8.5 months of age, tau pathology in the hippocampus was compared with that of untreated *PS19*^ΔTLR2^ and *PS19* mice by conducting immunohistochemistry with Tau-5 antibodies. Tau aggregation was monitored in both DG (**B**) and CA1 (**D**) neurons. Scale bars: 20 μm (left columns), 10 μm (right columns). (**C** and **E**) OD of tau expression was calculated relative to that in *nTg* mice. Two sections from each brain were used for the staining and quantitative analysis of tau expression, and the values obtained from each section are shown in the bar diagram. Images are shown at 20× and 40× magnifications. Total tau content in sarkosyl-soluble (**F**) and insoluble fractions (**H**) was assessed by Western blotting. The tau band densities obtained from the sarkosyl-soluble (**G**) and -insoluble fractions (**I**) was normalized to the loading control, actin, present in the soluble fraction. Arrows indicate the different isomers of tau, and the band near 70 kDa obtained from the sarkosyl-soluble fraction was considered for density analysis. Spatial learning and memory were tested by Barnes maze (**J**, heat map; **K**, error; **L**, latency). Statistical analyses were performed using 2-way ANOVA followed by Tukey’s multiple-comparison analysis. **P < 0.01 and ***P < 0.001 compared with the respective groups. Values are presented as mean ± SEM (*n* = 4 animals per group).

To confirm that the effect of wtTIDM was mediated via TLR2 in vivo, we prepared double-transgenic mice heterozygous for mutant tau and homozygous for TLR2-null mutation by breeding *PS19* mice with TLR2^–/–^ mice. These mice were designated as *PS19*^ΔTLR2^ and validated by genotyping (Figure 7A). First, 8.5-month-old *PS19* mice were compared with age-matched *PS19*^ΔTLR2^ mice in terms of glial activation, neuronal tau filament deposition, and the overall level of sarkosyl-soluble/insoluble tau. Immunohistochemical analysis of Iba1 showed a decrease in microglial activation in the hippocampus of *PS19*^ΔTLR2^ as compared with *PS19* mice (Supplemental Figure 15, A and C). Similarly, GFAP staining also indicated reduced astroglial activation in the hippocampus of *PS19*^ΔTLR2^ in comparison with *PS19* mice (Supplemental Figure 15, B and D). However, when 7-month-old *PS19*^ΔTLR2^ mice were treated with wtTIDM for 1.5 months, there was no further decrease in either microglial (Supplemental Figure 15, A and C) or astroglial (Supplemental Figure 15, B and D) activation.

Monitoring the status of NFTs also demonstrated that *PS19*^ΔTLR2^ mice had drastically decreased NFT formation in neuronal bodies present in both DG (Figure 7, B and C) and CA1 (Figure 7, D and E) compared with *PS19* mice. Furthermore, protein analysis revealed no significant change in the sarkosyl-soluble form of total tau between these 2 groups (Figure 7, F and G), whereas markedly less tau was found in the insoluble fraction of the *PS19*^ΔTLR2^ compared with *PS19* hippocampus (Figure 7, H and I). However, as with glial activation (Supplemental Figure 14), there was no further decrease in NFT level (Figure 7, B–E) or in the detergent-insoluble pathogenic tau level (Figure 7, F–I) compared with the untreated *PS19*^ΔTLR2^ mice. Similarly, Barnes maze test results also indicated attenuated impairment in spatial learning and memory in *PS19*^ΔTLR2^ compared with *PS19* mice (Figure 7, J–L). Again, although wtTIDM improved spatial learning and memory in *PS19* mice (Figure 6), it was incapable of doing so in *PS19*^ΔTLR2^ mice (Figure 7, J–L). These results suggest that in the absence of functional TLR2 protein, wtTIDM remained unable to decrease pathological NFT formation and improve cognitive functions.

### Proinflammatory cytokine induces tau expression in neurons via NF-κB.

**Figure 8:**
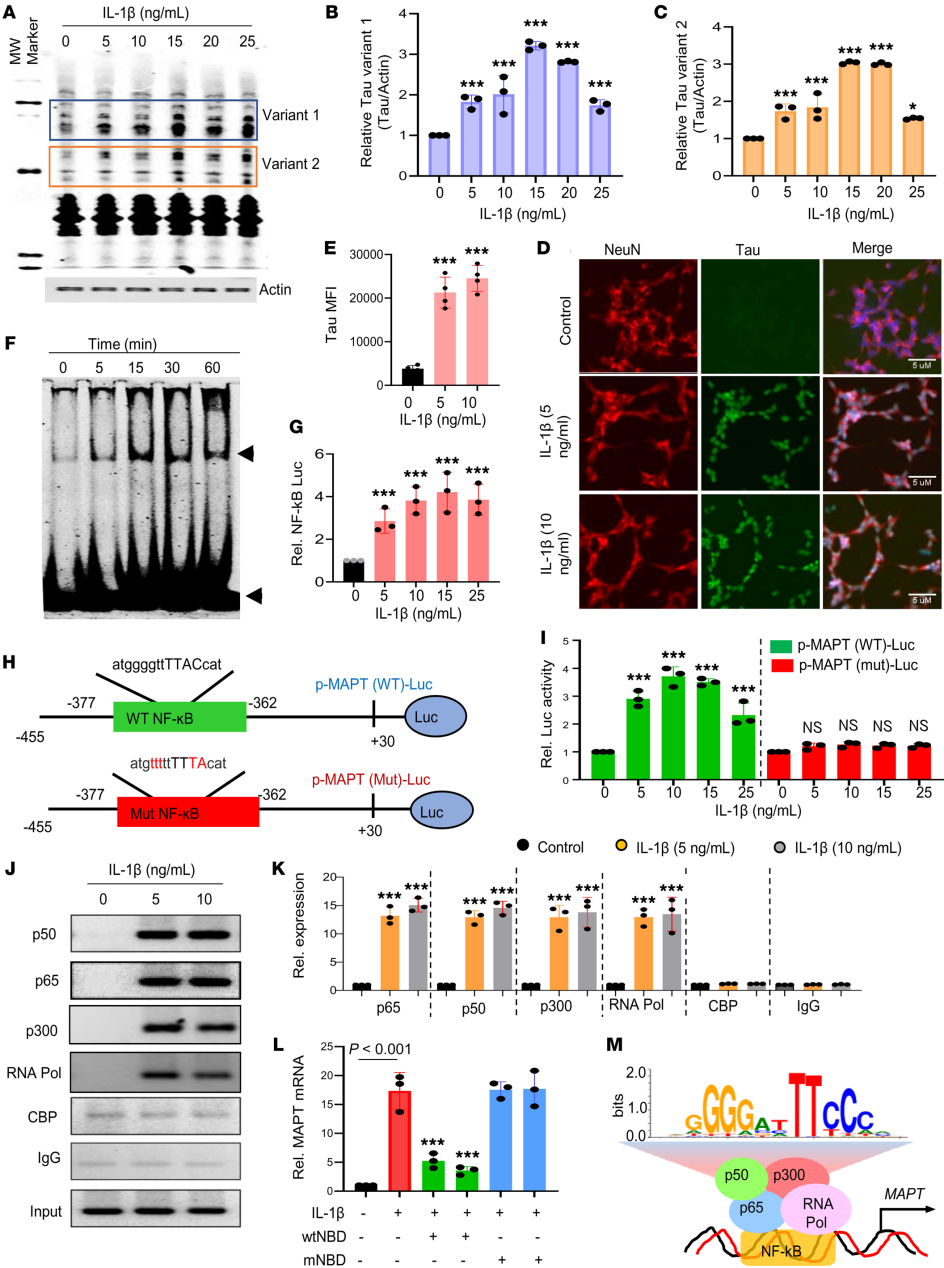
Inflammation induces neuronal tau expression via NF-κB activation. (**A**) Human SH-SY5Y cells were stimulated with different concentrations of IL-1β under serum-free conditions for 18 hours, then the level of total tau was monitored by Western blotting using Tau-5 antibody. Actin was run as a loading control. (**B** and **C**) Tau bands were scanned, and values (**B**, variant 1/actin; **C**, variant 2/actin) presented as relative (Rel.) to control. (**D**) Cells were double-labeled with Tau-5 and NeuN. (**E**) MFI of tau was measured by NIH ImageJ in 3 images of each of 3 different experiments. (**F**) After different periods of stimulation with IL-1β, the DNA-binding activity of NF-κB was monitored in nuclear extracts by EMSA. (**G**) Cells were transfected with PBIIx-Luc for 24 hours, followed by treatment with different concentrations of IL-1β for 4 hours, then luciferase assay in total cell extracts. (**H**) Map of the WT and mutated NF-κB sites of *MAPT* promoter luciferase constructs. (**I**) Cells were transfected with *pMAPT(WT)-Luc* and *pMAPT(mut)-Luc* for 24 hours, followed by treatment with IL-1β, and subjected to luciferase assay after 4 hours of stimulation. Cells were treated with IL-1β for 1 hour in serum-free medium, followed by ChIP analysis. Immunoprecipitated chromatin fragments were amplified by semiquantitative (**J**) and quantitative PCR (**K**) using primers mentioned in Methods. (**L**) Cells preincubated with either wtNBD peptide or mNBD peptide for 30 minutes were stimulated by IL-1β for 4 hours, followed by analysis of *MAPT* mRNAs by quantitative real-time PCR. (**M**) The schematic diagram showing a detailed map of promoter analysis of the *MAPT* gene. Results are the mean ± SD of 3 separate experiments. One-way ANOVA followed by Tukey’s multiple-comparison test was used for statistical analyses. *P < 0.05 and ***P < 0.001 versus control.

Our findings confirmed that TLR2 inhibition reduces both tau-mediated glial inflammation and formation of NFTs in neurons. It is still unclear how inflammatory molecules released from glial cells facilitate tau aggregation in neurons. To address this issue, we treated SH-SY5Y human neuroblastoma cells with IL-1β, a proinflammatory cytokine released by activated microglia and other cells, and monitored the protein expression of tau. Interestingly, we found that tau expression was elevated in SH-SY5Y cells with increasing doses of IL-1β (5–25 ng/ml) (Figure 8A). As human cells express a total of 6 alternatively spliced forms of tau, we have categorized these forms in 2 variants, where variant 1 represents the tau isoforms of higher molecular weight and variant 2 indicates the relatively lower-molecular-weight isoforms of tau; both of these variants were found to be increased following IL-1β exposure (Figure 8, B and C). IL-1β–induced tau upregulation was again validated by immunocytochemistry in SH-SY5Y cells with 2 doses of IL-1β (5 and 10 ng/mL) (Figure 8, D and E). IL-1β signaling is known to activate the transcription factor NF-κB. Therefore, we examined the involvement of NF-κB activation in inflammation-induced tau expression in neurons. The DNA-binding activity of NF-κB was enhanced after IL-1β treatment, as evidenced by the formation of a distinct and specific complex in a gel shift DNA-binding assay (Figure 8F). It led to an increase in transcriptional activity of NF-κB, as shown by luciferase activity from a PBIIx-Luc construct, with maximum activation seen at a concentration of 15 ng/mL (Figure 8G). Next, we searched the promoter region of tau using the MatInspector program and found a consensus binding site of NF-κB from 362 to 377 bp upstream of the transcription start site (Figure 8H). We cloned the tau promoter region containing the NF-κB–binding site into the PGL3 enhancer vector [*p-MAPT(WT)*]. We also mutated the core NF-κB–binding site and the mutated promoter construct [*p-MAPT(Mut)*] was cloned into the PGL3 vector. We observed that IL-1β significantly induced luciferase activity driven by the WT [*p-MAPT(WT)*], but not the mutated [*p-MAPT(Mut)*], tau promoter (Figure 8I). Furthermore, ChIP coupled with real-time PCR analyses was conducted to validate NF-κB–mediated transcriptional control of tau in SH-SY5Y cells. Classical NF-κB is a heterodimer of 2 subunits, p50 and p65, which were found to be highly recruited in the tau promoter following IL-1β treatment (Figure 8, J and K). As transcriptional activation requires association of histone acetyltransferases, we examined recruitment of CREB-binding protein (CBP) and p300, and found that IL-1β stimulation caused recruitment of p300, but not CBP, to the tau gene promoter; this coincided with enrichment of RNA polymerase (RNA Pol) to the tau promoter, resulting in transcriptional firing of the tau gene (Figure 8, J and K). Activation of NF-κB in neurons for inflammation-induced tau upregulation was specific, as inhibition of NF-κB by the WT NF-κB essential modifier (NEMO) binding domain (wtNBD) peptide markedly inhibited IL-1β–induced upregulation of tau gene expression in SH-SY5Y cells (Figure 8L).

To understand whether proinflammatory cytokine-induced upregulation of tau in primary human neurons also depends on NF-κB, we cultured primary human neural stem cells in Neurobasal medium containing 2% B27 and 1% antibiotic-antimycotic mixture (MilliporeSigma). After 9 days of culture, cells uniformly expressing MAP2 (Supplemental Figure 16B) were treated with NBD peptides and IL-1β. As in SH-SY5Y cells, IL-1β treatment led to substantial upregulation of *MAPT* mRNA in primary human neurons that was strongly inhibited by wtNBD, but not mNBD, peptide (Supplemental Figure 16A). Double-label immunofluorescence of MAP2 and tau also confirmed an increase in tau protein in primary human neurons with IL-1β challenge, which was suppressed by wtNBD, but not mNBD, peptide (Supplemental Figure 16, B and C). Collectively, these findings strongly suggest that inflammation triggers NF-κB activation complex in the tau promoter to induce transcriptional upregulation of tau in human neurons (Figure 8M).

### Fibrillar Aβ induces tau expression in neurons via NF-κB.

Development of Aβ pathology happens prior to the onset of tauopathy in human AD brains and it is also recapitulated in relevant animal models (26, 27). If NF-κB activation is required for upregulation of neuronal tau expression, it is possible that fibrillar Aβ also employs the NF-κB pathway to potentiate tau expression. To investigate this possibility, we treated SH-SY5Y cells with increasing doses of Aβ (0.5–2 μM), and interestingly found significant increases in tau variants in neurons (Supplemental Figure 17, A–C). Immunostaining of tau in SH-SY5Y cells further validated the increase in tau expression after Aβ treatment (Supplemental Figure 17, D and E). As with the inflammation-induced stimulation, we observed enhanced DNA binding of NF-κB following Aβ treatment, as revealed by the formation of a distinct and specific complex in a gel shift DNA-binding assay (Supplemental Figure 17F). Accordingly, enhanced transcriptional activity of NF-κB in Aβ-treated cells was confirmed by luciferase assay (Supplemental Figure 17G). Evidence for the specific involvement of NF-κB in the transcriptional upregulation of tau was strengthened by the finding that wtNBD, but not mNBD, treatment caused significant inhibition of Aβ-induced tau mRNA expression in neurons (Supplemental Figure 17H). Furthermore, Aβ significantly increased luciferase activity in cells harboring the WT tau promoter, but not the mutated tau promoter without the NF-κB–binding site (Supplemental Figure 17I). This indicates that Aβ-induced stimulation caused tau upregulation in neurons via NF-κB activation.

As NF-κB is shown to be the prime transcription factor for upregulation of tau, we attempted to examine whether stimulation of NF-κB by other canonical inducers of inflammation also increases neuronal tau expression. Therefore, SH-SY5Y cells were transfected with either WT [*p-MAPT(WT)*] or mutated [*p-MAPT(mut)*] tau promoter–driven reporter constructs and then stimulated with MPP^+^ (parkinsonian neurotoxin), HIV-I Tat (one of the etiological agents for HIV-associated neurocognitive disorder), gp120 (HIV-1 envelope glycoprotein), TNF-α (proinflammatory cytokine), poly IC (one of the etiological reagents for viral encephalopathy), flagellin (bacterial infection), and IFN-γ (Th1-released cytokine). The results showed an increase in luciferase activity from the *p-MAPT(WT)* promoter, but not from the *p-MAPT(mut)* promoter, following treatment with these inflammatory agents (Supplemental Figure 18, A–F) — except in the case of IFN-γ stimulation, which activated both the WT and mutated constructs (Supplemental Figure 18G), suggesting that IFN-γ might stimulate *MAPT* independently of NF-κB activation. More interestingly, when the same experiment was conducted in SH-SY5Y cells with tau PFF stimulation, a significant increase in luciferase activity was found in cells harboring the *p-MAPT(WT)* promoter, but not the *p-MAPT(mut)* promoter (Supplemental Figure 18H), highlighting that tau PFFs can directly activate intraneuronal tau expression via the NF-κB pathway.

## Discussion

The disease-related mutations of tau, including P301S and P301L, are known to cause reduced interaction of this protein with microtubules (28). Mutated tau has a higher tendency to self-aggregate and form paired helical filaments, which is dependent on its R2 and R3 regions at the C-terminus (29) and might also be dependent on heavy phosphorylation of certain residues (30, 31). Substantial aggregation of tau leads to progressive formation of NFTs, which hampers multiple cellular machineries, including ER, vesicle transport, autophagy, and mitochondrial functioning. As the excessive burden of tau progressively increases and spreads to other brain regions, the possibility arises that glial cells might also interfere in the pathogenesis. Ongoing research clearly demonstrates that microglia phagocytose extracellular tau (32) via interaction with CX3CR1 (33), and therefore, tau aggregates are also found to be localized in microglia in AD animals as well as in patients’ brains (34, 35). However, the efficiency of microglia in degrading tau oligomers or fibrils is still questionable, as addition of AD-derived tau has been shown to generate dystrophic microglia with swelling of lysosomes (12, 36). Moreover, it was still not clear whether tau aggregates activate microglia after being phagocytosed, or if this activation requires binding of tau to specific receptors on microglia. This necessitated identification of the mechanism behind tau fibril–induced glial inflammation.

Our present study unveils some major findings pertinent to tau fibril–induced glial activation. First, in vitro synthesized tau PFFs activated microglia specifically via the TLR2/MyD88, not the TLR4/MyD88, pathway. Second*,* postmortem AD brain–derived tau also required TLR2 for the activation of microglia. Third*,* activation of the TLR2/MyD88 pathway led to NF-κB–mediated transcriptional upregulation of major inflammatory genes. The involvement of TLR2 in inducing glial inflammation was evidenced by the fact that wtTIDM, which specifically targets TLR2-MyD88 (16), significantly blocked this effect. Fourth*,* wtTIDM-mediated suppression of inflammation was recapitulated in *PS19* mouse brain, further emphasizing the involvement of TLR2 in generating glial inflammation in tauopathy brain. Our previous report clearly showed availability of TIDM peptides in the hippocampus following nasal administration (16). Fifth*,* inhibition of glial inflammation coincided with reduced NFT formation in the hippocampus, suggesting that inflammation is pivotal for developing tau-associated neuronal pathology. Sixth*,* attenuated tau accumulation in neurons resulted in better synaptic functioning of the hippocampus, ultimately leading to improved learning and memory consolidation ability of *PS19* animals. Seventh*,* the unaltered effect of wtTIDM on glial activation and tau pathology in TLR2 ablated *PS19* animals proves that the effect of wtTIDM is specific to TLR2 in vivo. Last*,* our study provides the evidence that inflammatory molecules activate tau expression in SH-SY5Y neuronal cells and primary human neurons through NF-κB dependent manner.

Synthetic fibrillar tau closely mimics features of tau fibrils obtained from human AD brains (37). Injection of tau PFFs in entorhinal cortex not only recapitulates the signature mode of its propagation toward hippocampus, but also forms more complex proteinase-resistant aggregates that are immunostained by TG3, a conformation-specific phospho-tau mAb, by anti-acetylated tau K280 antibody and also strongly stained for Thio-S (15, 38, 39). It explains the reason behind considering synthetic tau PFFs for studying glial activation in vitro. Efforts made by previous studies have demonstrated that tau, in monomeric, oligomeric and fibrillar forms activates microglia. However, monomeric tau-induced inflammation was not found to be as enormous as fibrillar tau as the extent of upregulation of inflammatory molecules was much less, whereas the dose required to induce inflammation was much higher compared with the PFFs used by us and also by another study (40, 41). The existing report also highlighted the involvement of p38 MAPK in mediating tau-induced inflammation. It has to be considered that kinases activated by tau exposure are present in the cytosol, whereas primary interaction of microglia with extracellular protein aggregates must involve any receptor protein in the cell membrane. In that context, TLR2 has not been hypothesized in previous studies to be a receptor protein of tau PFFs. Our findings on tau-mediated TLR2 activation fit well with the observations in previous studies that TLR2 activation can lead to activation of different kinases including p38 MAPK and can trigger microglial phagocytosis (42, 43). In addition, these reports did not show the crucial involvement of canonical NF-κB activation in inducing inflammatory genes in PFF-exposed microglia, which is revealed in the present study. There was a possibility that PFF-induced NF-κB activation might also result from activation of other TLRs, specifically TLR4, which is known to interact with different oligomeric aggregated forms of proteins including Aβ (43, 44). However, the absence of induction of inflammatory genes in TLR2^–/–^, but not in TLR4^–/–^ microglia, and also wtTIDM-sensitive suppression of inflammation in PFF-exposed microglia convincingly establishes the role of TLR2/MyD88 in carrying out the inflammatory effect of tau fibrils in microglia.

The enhanced level of TLR2 and MyD88 in glial cells of 7-month-old *PS19* brains provides a sharp indication that upregulation of these proteins happens concurrently with gliosis. Most importantly, these events paralleled NFT formation, as *PS19* mice of this age had previously been shown to develop marked tau and phospho-tau deposition (45). It can be anticipated that in the presence of prominent tau pathology, activation of TLR2 and NF-κB in resident microglia leads to secretion of inflammatory factors that further augment gliosis in the brain. It is also noteworthy that astroglial upregulation of TLR2 and MyD88 under pathological conditions can lead to astroglial inflammation, whereas microglial activation can further boost generation of inflammatory molecules by astrocytes (46). Evidence for TLR2-dependent inflammation in tauopathy was reinforced, as nasal administration of wtTIDM caused downregulation of microgliosis, astrogliosis, and inflammation in the brain. Moreover, reduction of neuronal tau deposition and NFT formation in the hippocampus in wtTIDM-induced mice established a strong connection between glial inflammation and tau aggregation in vivo. On the other hand, synapse loss in hippocampal neurons precedes NFT formation and neuronal death in *PS19* mice (15, 47). In that context, attenuated loss of synaptic integrity by wtTIDM was proven by the increased level of PSD95 protein on CA1 neurons. PSD95 is greatly implicated in scaffolding of multiple receptor proteins activated by the excitatory neurotransmitter glutamate and in promoting glutamate receptor–mediated Ca influx required for memory formation (48, 49). Upregulation of PSD95 in wtTIDM-treated mice corrected the Ca influx in hippocampal cells and retained synaptic plasticity in *PS19* animals. This finding demonstrates the indirect neuroprotective effect of TLR2 inhibition on overall functioning of the hippocampus; the effect was further replicated in the behavioral studies, in which wtTIDM-treated animals displayed better learning capability as well as retention of spatial memory. That participation of TLR2 is crucial in tau pathology was strongly supported by the finding that TLR2 ablation significantly abrogated formation of insoluble tau deposition in neurons. Furthermore, the finding that wtTIDM treatment of TLR2-ablated *PS19* animals did not reduce tau pathology clearly suggested that wtTIDM peptide required TLR2 for exerting its neuroprotective effect.

Another important finding in this study is that the tau promoter contained a potential NF-κB–binding site. As a majority of the inflammatory factors employ the NF-κB pathway to activate target genes, the presence of this consensus sequence in the tau promoter clearly exhibited the bidirectional relationship between inflammation and tauopathy. More interestingly, as neurons express TLR2 on the cell membrane and TLR2 expression was also found to be increased in neurons under degenerative conditions (50), there is a possibility that extracellular tau can even activate the TLR2/MyD88/NF-κB pathway in neurons to activate tau transcription, and that facilitates irreversible tau generation and accumulation in neurons. Recently, a report elaborated the role of microglial NF-κB in tau-mediated inflammation and tau spreading (51). Our report adds knowledge to this subject by demonstrating that not only tau-mediated glial inflammation but also inflammation-mediated tau exacerbation in neurons was dependent on the NF-κB pathway. Therefore, these two events can be seen a double-edged sword: both aspects can be controlled by targeting the TLR2/MyD88/NF-κB pathway.

In the last decade, numerous studies have demonstrated antiinflammatory and neuroprotective effects of different pharmacological molecules in tauopathy brains (23), but in the absence of proper knowledge about tau-induced receptor activation in glial cells, these approaches might face several challenges. This study reveals the TLR2/MyD88/NF-κB pathway as the potential link between the signature prerequisite events of progressive neurodegeneration in tauopathy brains: tau aggregation and glial inflammation. Therefore, blocking TLR2/MyD88/NF-κB–mediated inflammation with the synthetic peptide wtTIDM holds potential therapeutic value against AD, PSP, FTD, CBD, and other tauopathies.

## Methods

### Reagents.

DMEM was purchased from Mediatech, and FBS was obtained from Atlas Biologicals. Antibiotic-antimycotic was purchased from MilliporeSigma. Recombinant human Tau-441 was purchased from AnaSpec. Primary antibodies used in the study are listed in Supplemental Table 1. Cy2- and Cy5-conjugated antibodies and fluorophore-tagged secondary antibodies were obtained from Jackson ImmunoResearch Laboratories Inc. AD brain–derived insoluble tau was provided by Virginia M.-Y. Lee, University of Pennsylvania School of Medicine, Philadelphia, Pennsylvania, USA.

### Animals.

Adult *C57BL6*, *TLR2^–/–^* (*B6.129-Tlr2^tm1Kir^/J*), *TLR4^–/–^* (*B6(Cg)-Tlr4tm1.2Karp/J*), and *PS19MAPT* (B6;C3-Tg(Prnp-MAPT*P301S)PS19Vle/J) mice were purchased from The Jackson Laboratory.

### Cell culture and isolation of primary mouse microglia.

Primary microglia were isolated from mixed glial cultures as described by us previously (52, 53). BV-2 murine microglial cells (a gift from V. Bocchini, University of Perugia, Perugia, Italy) was maintained in DMEM/F12 medium containing 10% FBS at 37°C in the incubator.

### Culturing human neurons.

Culturing of human neurons was performed as described previously (54, 55). For details, see Supplemental Methods.

### TIDM peptides.

TIDM peptides (>99% pure) (16, 50) were synthesized in the custom peptide synthesis facility of GenScript. TIDM peptides contained the antennapedia homeodomain (lower case) and 6-amino-acid-long MyD88 (upper case) segments: WT TIDM: drqikiwfqnrrmkwkkPGAHQK; mTIDM: drqikiwfqnrrmkwkkPGWHQD. Positions of mutations are underlined.

### Intranasal treatment of animals with TIDM peptides.

Intranasal treatment of mice with TIDM peptides was performed as described previously (16, 50). For details, see Supplemental Methods.

### Preparation and validation of tau PFFs.

Full-length human tau monomers (2N4R tau) were solubilized in 0.1 M acetate buffer (pH 7.4) at a concentration of 44 μM to prepare the tau stock. During fibrillation, 22 μM tau along with heparin (1:4 molar ratio) was rotated continuously in a rotary shaker at 500 rpm at 37°C for 7 days (37). Next, the solution containing tau and heparin was centrifuged at 100,000*g* for 30 minutes, at 4°C to precipitate the tau fibrils. The supernatant was discarded, and the pellet containing fibrils was solubilized in an equal volume of acetate buffer. The PFFs were characterized by electron microscopy (EM). For EM imaging, 1 μL stock solution was diluted in 10 μL PBS, and this solution was adsorbed to a 300-mesh copper, Formvar-coated EM grid, washed, and stained with 1% uranyl acetate, and the grid was allowed to dry for 15–20 minutes. Imaging was performed at 100,000× magnification using a JEOL JEM-1220 transmission electron microscope (operating at 80 kV). Digital micrographs were acquired using an Erlangshen ES1000W model 785 CCD camera and DigitalMicrograph software (version 1.7).

### Endotoxin assay.

See Supplemental Methods for details on the endotoxin assay.

### Tissue lysate preparation.

Tissue lysate was prepared as described previously (16, 50). For details, see Supplemental Methods.

### Western blotting.

Western blotting was performed as previously described (56, 57). For details, see Supplemental Methods.

### Immunostaining.

Immunostaining was carried out as described previously (58, 59). For details, see Supplemental Methods.

### Real-time PCR.

Total RNA was isolated from primary microglia using the QIAGEN RNeasy kit following the manufacturer’s protocol. The isolated RNA was reverse transcribed into cDNA, and real-time PCR was performed using the following the primers: mouse iNOS — sense 5′-CCCTTCCGAAGTTTCTGGCAGCAGC-3′, antisense 5′-GGCTGTCAGAGCCTCGTGGCTTTGG-3′; mouse IL-1β — sense: 5′-GGATATGGAGCAACAAGTGG-3′, antisense 5′-ATGTACCAGTTGGGGAACT-3′; mouse TNF-α — sense 5′-TTCTGTCTACTGAACTTCGGGGTGATCGGTCC-3′, antisense 5′-GTATGAGATAGCAAATCGGCTGACGGTGTGGG-3′; human MAPT — sense 5′-ACTGGCATCTCTGGAGTGTGTG-3′, antisense 5′-GCAGCTACAAGCTAGGGTGCAAG-3′; mouse GAPDH — sense: 5′-GGTGAAGGTCGGTGTGAACG-3′, antisense 5′-TTGGCTCCACCCTTCAAGGTG-3′. Real-time PCR was carried out in a QuantStudio 3 detection system (Thermo Fisher Scientific) using the SYBR green real-time kit obtained from Quantabio. Cycle threshold crossing point(*C_t_*) values for each sample were obtained from the software, and data were analyzed using the 2^−ΔΔCt^ method as described previously (60, 61).

### IP.

IP was performed as described previously (16, 50). For details, see Supplemental Methods.

### ChIP assay.

Recruitment of NF-κB to the *MAPT* gene promoter was determined by ChIP assay as described earlier (62, 63). For details, see Supplemental Methods.

### EMSA.

EMSA was carried out as described previously (16, 61, 63). For details, see Supplemental Methods.

### Construction of the human MAPT promoter–driven reporter construct.

Human genomic DNA isolated from primary human neurons was used as the template during PCR. The 5′-flanking sequence of human *MAPT* (−455/+30) gene was isolated by PCR. Primers were designed from GenBank sequences as follows: *MAPT* — sense 5′-acgcgt CTCCTGCCTCAGCCTCCCCAGTAGC-3′, antisense 5′-gagctcTCTTCCATCACTTCGAACTCCTGGC-3′. While the sense primer was tagged with a *MluI* restriction enzyme site, the antisense primer was tagged with a *XhoI* restriction endonuclease site. PCR was performed using an Advantage-2 PCR kit (Clontech) according to the manufacturer’s instructions. The resulting fragments were gel-purified and ligated into the pGEM-T Easy vector (Promega). These fragments were further subcloned into the PGL3 Enhancer vector after digestion with the corresponding restriction enzymes and verification by sequencing (ACGT Inc. DNA Sequencing Services).

### Cloning of human MAPT promoter and site-directed mutagenesis.

Site-directed mutagenesis was performed as described earlier (50, 62, 64) by using a site-directed mutagenesis kit (Stratagene). Two primers in opposite orientations were used to amplify the mutated plasmid in a single PCR. The primer sequence for the mutated promoter site was as follows: sense, 5′-GTATTTTTAGTAGAGATGTTTTTACATGTTGGCCAGG-3′, and antisense, 5′-CCTGGCCAACATGTAAAAACATCTCTAAAAAATAC-3′. The PCR product was precipitated with ethanol and then phosphorylated by T4 kinase. The phosphorylated fragment was self-ligated by T4 DNA ligase and digested with the restriction enzyme *DpnI* to eliminate the nonmutated template. The mutated plasmid was cloned and amplified in *E*. *coli* (DH5-α strain)–competent cells.

### Luciferase assay.

The luciferase assay was performed as described previously (65, 66). For details, see Supplemental Methods.

### Ca assay.

Ca influx in hippocampal slices was measured as described earlier (67, 68). For details, see Supplemental Methods.

### Behavioral tests.

To determine cognitive function and movement abilities in mice, 4 major kinds of behavioral tests (Barnes maze, NORT, open field test, and rotarod test) were performed as described earlier (56, 58, 64, 67, 68). For details, see Supplemental Methods.

### Statistics.

Statistical analyses were performed using GraphPad Prism v9.0. Values are expressed as mean ± SD for data obtained from cellular studies and mean ± SEM for animal experiments. Statistical comparisons between 2 different samples were conducted by using unpaired 2-tailed *t* test. One-way ANOVA followed by Tukey’s multiple-comparison test was performed for statistical analyses among multiple groups. Two-way ANOVA was used for comparing more than 1 parameter among different groups. The criterion for statistical significance was *P* < 0.05.

### Study approval.

Animal housing, maintenance, and experiments were performed following the guidelines provided by the NIH and were approved by the IACUC (protocol 21-044) of the Rush University Medical Center.

### Data availability.

No new code was generated in this study; all analyses were performed using existing packages. Values for all data points in graphs are reported in the Supporting Data Values file.

## Author contributions

KP conceived the original idea, supervised the project, acquired the funding, and edited the final version of the manuscript. DD, MJ, and KP designed the study. DD, MJ, RKP, MM, SR, and SD performed the research. DD, MJ, RKP, MM, SR, SD, and KP analyzed the data. DD wrote the first draft of the manuscript.

## Supplementary Material

Supplemental data

Supporting data values

## Version 1 08/08/2023

In-Press Preview

## Version 2 09/15/2023

Electronic publication
