# Supplementary material for: Tau fibrils induce glial inflammation and neuropathology via TLR2 in Alzheimer’s disease–related mouse models
Source: J Clin Invest. 2023 Sep 15;133(18):e161987. doi: 10.1172/JCI161987 (PMC10503811; doi:10.1172/JCI161987)
Supplement: Supplemental data [file jci-133-161987-s197.pdf]

Tau fibrils induce glial inflammation and neuropathology via TLR2 in Alzheimer's disease-related mouse models

Debashis Dutta[1,*], Malabendu Jana[1,*], Ramesh Kumar Paidi[1], Moumita Majumder[1], Sumita Raha[1], Sridevi Dasarathi[1], and Kalipada Pahan[1,2]

[1]Department of Neurological Sciences, Rush University Medical Center, Chicago, USA;
[2]Division of Research and Development, Jesse Brown Veterans Affairs Medical Center, Chicago, USA

[*] First two authors have equal contribution to the work.

Conflict of interest: None

Running title: Role of TLR2 in tauopathy

Address correspondence to:
Kalipada Pahan, Ph.D.
Department of Neurological Sciences
Rush University Medical Center
1735 West Harrison St, Suite Cohn 310
Chicago, IL 60612
Tel: (312) 563-3592
Fax: (312) 563-3571
Email: Kalipada_Pahan@rush.edu

Supplemental methods:
Culturing human neurons
Neurons and neural stem cells (NSCs) were prepared from 11- to 17-week-old human fetal brains obtained from the Human Embryology Laboratory (University of Washington, Seattle, WA) in 2007 following approval (ORA # 06080303) by the Institutional Review Board of the Rush University Medical Center (1, 2). Some of these cells were kept frozen in liquid nitrogen. Although neurons did not survive after thawing and re-culturing after about 15 years of freezing, NSCs survived, which were allowed to grow in Neurobasal medium containing 2% B27 and 1% antibiotic-antimycotic mixture (Sigma) for differentiation into neurons. After 9 d of culture, immunofluorescence analysis showed that these cells uniformly expressed MAP2 (Fig. S16b).
Intranasal treatment of animals with TIDM peptides
It was performed as described before (3, 4). Briefly, the wtTIDM and mTIDM peptides were solubilized in normal saline in such a way so that 0.1 mg/kg of body weight TIDM peptide is contained in 5 µl of saline. Then 2.5 µl of TIDM solution was administered in mice through each nostril every day. Mice were hold in supine condition while administering TIDM solutions. Intranasal treatment was started for *PS19* animals at the age of 7 months. After 30 d of treatment, when the animals reached 8 months of age, behavioral experiments including training and acquisition were conducted for the next two weeks and then the experimental mice were sacrificed. Therefore, the total treatment of TIDM peptides was continued for 45 days.
Endotoxin assay
Level of endotoxin was measured in tau PFF by using the ToxinSensor™ Endotoxin Detection System kit (Genscript, Piscataway, NJ) following manufacturer's protocol. Briefly, all the reagents were reconstituted and 100 µl chromogenic substrate solution were added to each reaction vial followed by incubation at 37^0C for 6 min. Then 500 µl stop solution was added to each vial followed by addition of 500 µl color-stabilizer #2 to each vial. Finally, 500 µl color-stabilizer #3 was added to each vial followed by gentle mixing and reading the absorbance of resultant solution at 545 nm in a microplate reader.
Tissue lysate preparation
Hippocampal tissues were taken in 10 (v/v) of extraction buffer containing 10 mM Tris-HCl (pH=7.4), 0.8 M NaCl, 10% sucrose, 1 mM EGTA, 2% sarkosyl, and protease/phosphatase inhibitors, sonicated for 20-30 s and incubated in 4^oC for 30 min. The homogenate was centrifuged at 20000 xg for 10 min at 4^oC. Supernatant was taken into fresh tube and then ultracentrifuged at 100000 xg for 30 min at 4^oC. The supernatant was collected and marked as the sarkosyl soluble fraction. The pellet was further washed by ultracentrifugation with 0.5 ml sterile water. The pellet was solubilised in 50 mM Tris-HCl (pH=7.4). Protein concentration was measured from both the sarkosyl soluble and insoluble fractions and samples were prepared for Western blotting in SDS containing sample buffer (5).
Total tissue lysate was obtained by dissolving the tissue in RIPA buffer (50 mM Tris, pH 8.0,150 mM NaCl, 1% Nonidet P-40, 1% SDS, 0.5% sodium-deoxycholate) with complete protease and phosphatase inhibitor cocktails. Tissues were sonicated for 20-30 s and the homogenate was centrifuged at 17,500 xg for15 min at 4^oC, and the resulting supernatant was collected.
Western Blotting
Western blotting was performed as previously described (6, 7). Briefly, equal amounts of proteins were electrophoresed in 8% (for monitoring tau level) or 10% SDS-PAGE and transferred onto nitrocellulose membrane. The blot was probed with primary antibodies used at particular dilutions

overnight at 4°C (Supplementary Table 1). Next day primary antibodies were removed and the blots were washed with phosphate buffer saline containing 0.1% Tween-20 (PBST) and then probed with corresponding infrared fluorophore-tagged secondary antibodies (1:10,000; Jackson Immuno-Research) and kept at room temperature (RT) for 1 h. Finally, blots were scanned with an Odyssey infrared scanner (Li-COR, Lincoln, NE). Band intensities were quantified using ImageJ software (6, 7).

Immunostaining

Immunostaining was performed as described earlier (8, 9). Briefly, animals were perfused with 4% paraformaldehyde, and the brains kept in 30% sucrose solution at 4°C. Using a cryotome, 30-µm coronal sections were cut from the hippocampal region of the brain and processed for immunostaining. Immunostaining was performed using the procedure described earlier with certain modifications(8, 9). In case of tau and phospho-tau staining, tissue sections were permeabilized with 0.1% of Triton X-100 and blocked with PBS containing 4% normal horse serum and 2% BSA for 1 h. Primary antibodies made in 2% BSA was added onto sections and incubated overnight at 4°C. Next day, following incubation with biotin-tagged secondary antibodies, samples were kept in Vectastain A and B mixture solution at RT. Sections were developed by 3,3'-diaminodenzidine (DAB) solution containing peroxide. Immunofluorescence of tissue and cell samples was conducted using the protocol mentioned by our earlier studies. The experimental cell and tissue samples were imaged under either Olympus BX41 fluorescence microscope or Zeiss confocal microscope using Zen 2012 software (Zeiss LSM 780, Carl Zeiss, Jena, Germany). Mean fluorescence intensity (MFI) and counting of target proteins or cells were performed using ImageJ. Intensity of tau and phospho-tau DAB staining was quantified using Fiji (ImageJ2). All figures were deconvoluted to achieve H-DAB stained images and then each cell was outlined and the intensity was measured by the Analyze-measure option provided in the software (6). Darker staining indicates lower mean value. The mean value of white is considered to be highest (255), and therefore the formula used for calculating relative O.D. is $\log_{10}$(255/mean of each cell).

Immunoprecipitation (IP)

BV-2 cells were treated with 10 µM of wtTIDM or mTIDM. After 30 min of TIDM treatment, tau PFF (25 nM) was added to the cells and incubated for 2 h. Following the incubation period, cells were scraped and lysed in RIPA buffer. The cell homogenate was centrifuged at 17,500 xgfor15 min 4°C, supernatant was collected and protein was estimated using the BCA method. The cell lysate was immunoprecipitated with 2 µg of anti-MyD88 or normal IgG (Santa Cruz Biotechnology) overnight at 4°C, followed by incubation with protein A-agarose for 4 h at 4°C. Protein A-agarose-antigen-antibody complexes were collected by centrifugation at 10000 xg for 1 min at 4 °C. The pellets were washed for 3-4 times with 1 ml of IP buffer containing 20 mM Tris-HCl (pH 8.0), 137 mM NaCl, 2 mM EDTA, 1% Nonidet P-40, 10% glycerol and protease inhibitor cocktails for 20 min each time at 4°C. Bound proteins were resolved by SDS-PAGE, followed by Western blotting with the anti-TLR2 (1:1000, Abcam) and/or anti-TLR4 (1:1000, Abcam) antibodies. Input from each sample was also run in the Western blotting (4).

Chromatin immunoprecipitation (ChIP) assay

Recruitment of NF-κB to *MAPT* gene promoter was determined by ChIP assay as described earlier (10, 11). Briefly, SH-SY5Y cells were treated with IL-1β under serum free conditions and after 1 h of stimulation, cells were fixed by adding formaldehyde (1% final concentration), and cross-linked adducts were resuspended and sonicated. ChIP was performed on the cell lysate by overnight incubation at 4°C with 2 µg of anti-p65, anti-p50, anti-CBP, anti-RNA Polymerase II,

or anti-p300 antibodies followed by incubation with protein G agarose (Santa Cruz Biotechnology) for 2 h. The beads were washed and incubated with elution buffer. To reverse the cross-linking and purify the DNA, precipitates were incubated in a 65°C incubator overnight and digested with proteinase K. DNA samples were then purified, precipitated, and precipitate were washed with 75% ethanol, air-dried, and resuspended in TE buffer. Following primers were used for amplification of chromatin fragment (200 bp) of human *MAPT* gene.

Sense: 5'- CTCCTGCCTCAGCCTCCCCAGTAGC -3'

Antisense: 5'- CAGGTGACGGCCAGGCATGGTGGCT -3'.

Electrophoretic mobility shift assay (EMSA)

Nuclear extracts were prepared, and EMSA was performed after 1 h of PFF treatment in BV2 cells following the method described previously with some modifications. Briefly, IR Dye end-labelled oligonucleotides containing the consensus binding sequence for NF-κB were purchased from Licor Biosciences. Six micrograms of nuclear extract was incubated with binding buffer and with infrared-labelled probe for 20 min. Subsequently, samples were separated on a 6% polyacrylamide gel in 0.25× TBE buffer (Tris borate-EDTA) and analysed by the Odyssey Infrared Imaging System (LI-COR Biosciences) (4, 11, 12).

Luciferase Assay

Cells plated at 60-70% confluency were transfected with either pNF-κB-Luc or PBIIX-Luc construct using Lipofectamine plus (Life Technologies). Following 24 h of transfection, cells were incubated with wtTIDM or mTIDM (10 µM) for 1 h and then exposed to 25 nM of tau PFF under serum free conditions for 2 h. Luciferase activities were analysed in cell extracts using the Luciferase Assay System kit (Promega) in a TD-20/20 Luminometer (Turner Designs) as described previously (13, 14).

Calcium Assay

Ca influx in hippocampal slices was measured as described earlier (15, 16). Briefly, experimental animals were rapidly perfused with ice-cold PBS, brain was taken out and Dorsoventral slices of the hippocampus were cut at a thickness of 100 µm using an adult mouse brain slicer matrix with 1.0-mm coronal section slice intervals. The slices were immediately kept in ice-cold solution (24.56 g of sucrose, 0.9008 g of dextrose, 0.0881 g of ascorbate, 0.1650 g of sodium pyruvate, and 0.2703 g of myo-inositol in 500 mL of distilled water) saturated with 5% CO2 and 95% O2 gas mixture. Slices were then carefully transferred into Fluo-4 dye containing reaction buffer. The reaction buffer was prepared before the making of brain slices using 10 mL of artificial CSF (119 mM NaCl, 26.2 mM $NaHCO_3$, 2.5 mM KCl, 1 mM NaH_2PO_4, 1.3 mM $MgCl_2$, 10 mM glucose saturated with 5% CO2, and 95% O2, followed by the addition of 2.5 mM $CaCl_2$ added to a bottle of Fluo-4 dye, and 250 mM probenecid. Before the transfer of slices, a flat-bottom 96-well plate (BD Falcon; catalog #323519) was loaded with 50 µL of reaction buffer per well and kept in a dark place. One individual slice was placed in each well loaded with reaction buffer, and the plate was wrapped with aluminum foil and kept at 37 °C for 20 min. Then fluorescence excitation and emission spectra were recorded in a PerkinElmer VICTOR X2 luminescence spectrometer in the presence of NMDA (50 µM) and AMPA (50 µM). The recording was performed with 300 repeats at 0.1-ms intervals.

Behavioral tests

Barnes maze

During the training phase, mice were placed on the middle of the Barnes maze arena 10 min daily for consecutive 2 days. The arena was equipped with high wattage light to provide enough heat and motivation to the animals to find out the target hole containing the food pellet. Animals were

given rest for one day and on the 4th day the experiment was performed. Animals were food-deprived for overnight and during the test each animal was placed in the arena for 5 min and the performance was captured using the Noldus system. Memory of animals was analysed based on the parameters such as time taken to reach the goal box and number of errors made by each animal before reaching the goal box (15, 16).

NORT

NORT was conducted on a single day for the experimental animals. Two familiar objects were placed on opposite sides in the arena, which was under the coverage of a camera linked to Noldus system. A mouse was first placed into the arena and kept for 5-10 min so that the mouse becomes familiarize with the given objects. Then the mouse was withdrawn from the arena and kept back in the cage. One of the objects was replaced with a new object and after 20 min of the initial exposure, the same mouse was again brought to the arena. Time spent by the mouse on the novel object and the familiar object for the next 5 min was recorded by the software. This protocol was followed for each experimental mouse (15, 16).

Open field test

Locomotion was monitored with a camera linked to the Noldus system and the EthoVisionXT software (Netherlands). The instrument records the overall movement abilities of the animals including total distance moved, velocity, total moving time, resting time, center frequency and frequencies of movement and rest. Before the test, mice were placed inside the open field arena for 10 min daily for 2 consecutive days to train them and record their baseline values. Next day the mice were given rest and the on the following day each mouse was taken from the cage and gently placed in the middle of the open field arena. After releasing the animal, data acquisition was started by the software for the next 5 min and the parameters related to the locomotor activities were collected by the software (15-17).

Rotarod test

Mice were first acclimatized on the rotarod instrument for 5-10 min daily for consecutive two days. After 2 days of training, mice were placed on the rotating rod, which rotates with an accelerating speed of 4-40 rpm. The experiment was ended if the animal either slips from the rotating rod to the base of the instrument or just holds the rod to turn reverse without rotating against the direction of rotating rod (6, 8).

References to supplemental methods:

1. Jana A, and Pahan K. Fibrillar amyloid-beta-activated human astroglia kill primary human neurons via neutral sphingomyelinase: implications for Alzheimer's disease. *J Neurosci.* 2010;30(38):12676-89.
2. Jana M, Jana A, Pal U, and Pahan K. A simplified method for isolating highly purified neurons, oligodendrocytes, astrocytes, and microglia from the same human fetal brain tissue. *Neurochem Res.* 2007;32(12):2015-22.
3. Dutta D, Jana M, Majumder M, Mondal S, Roy A, and Pahan K. Selective targeting of the TLR2/MyD88/NF-kappaB pathway reduces alpha-synuclein spreading in vitro and in vivo. *Nat Commun.* 2021;12(1):5382.
4. Rangasamy SB, Jana M, Roy A, Corbett GT, Kundu M, Chandra S, Mondal S, Dasarathi S, Mufson EJ, Mishra RK, et al. Selective disruption of TLR2-MyD88 interaction inhibits inflammation and attenuates Alzheimer's pathology. *J Clin Invest.* 2018;128(10):4297-312.

5. He Z, McBride JD, Xu H, Changolkar L, Kim SJ, Zhang B, Narasimhan S, Gibbons GS, Guo JL, Kozak M, et al. Transmission of tauopathy strains is independent of their isoform composition. *Nat Commun.* 2020;11(1):7.
6. Dutta D, Majumder M, Paidi RK, and Pahan K. Alleviation of Huntington pathology in mice by oral administration of food additive glyceryl tribenzoate. *Neurobiol Dis.* 2021;153(105318.
7. Khasnavis S, and Pahan K. Cinnamon treatment upregulates neuroprotective proteins Parkin and DJ-1 and protects dopaminergic neurons in a mouse model of Parkinson's disease. *J Neuroimmune Pharmacol.* 2014;9(4):569-81.
8. Dutta D, Kundu M, Mondal S, Roy A, Ruehl S, Hall DA, and Pahan K. RANTES-induced invasion of Th17 cells into substantia nigra potentiates dopaminergic cell loss in MPTP mouse model of Parkinson's disease. *Neurobiol Dis.* 2019;132(104575.
9. Jana A, Modi KK, Roy A, Anderson JA, van Breemen RB, and Pahan K. Up-regulation of neurotrophic factors by cinnamon and its metabolite sodium benzoate: therapeutic implications for neurodegenerative disorders. *J Neuroimmune Pharmacol.* 2013;8(3):739-55.
10. Ghosh A, Jana M, Modi K, Gonzalez FJ, Sims KB, Berry-Kravis E, and Pahan K. Activation of peroxisome proliferator-activated receptor alpha induces lysosomal biogenesis in brain cells: implications for lysosomal storage disorders. *J Biol Chem.* 2015;290(16):10309-24.
11. Paidi RK, Jana M, Mishra RK, Dutta D, and Pahan K. Selective Inhibition of the Interaction between SARS-CoV-2 Spike S1 and ACE2 by SPIDAR Peptide Induces Anti-Inflammatory Therapeutic Responses. *J Immunol.* 2021;207(10):2521-33.
12. Paidi RK, Jana M, Mishra RK, Dutta D, Raha S, and Pahan K. ACE-2-interacting Domain of SARS-CoV-2 (AIDS) Peptide Suppresses Inflammation to Reduce Fever and Protect Lungs and Heart in Mice: Implications for COVID-19 Therapy. *J Neuroimmune Pharmacol.* 2021.
13. Pahan K, Liu X, McKinney MJ, Wood C, Sheikh FG, and Raymond JR. Expression of a dominant-negative mutant of p21(ras) inhibits induction of nitric oxide synthase and activation of nuclear factor-kappaB in primary astrocytes. *J Neurochem.* 2000;74(6):2288-95.
14. Pahan K, Jana M, Liu X, Taylor BS, Wood C, and Fischer SM. Gemfibrozil, a lipid-lowering drug, inhibits the induction of nitric-oxide synthase in human astrocytes. *J Biol Chem.* 2002;277(48):45984-91.
15. Patel D, Roy A, Kundu M, Jana M, Luan CH, Gonzalez FJ, and Pahan K. Aspirin binds to PPARalpha to stimulate hippocampal plasticity and protect memory. *Proc Natl Acad Sci U S A.* 2018;115(31):E7408-E17.
16. Patel D, Roy A, Raha S, Kundu M, Gonzalez FJ, and Pahan K. Upregulation of BDNF and hippocampal functions by a hippocampal ligand of PPARalpha. *JCI Insight.* 2020;5(10).
17. Roy A, Jana M, Corbett GT, Ramaswamy S, Kordower JH, Gonzalez FJ, and Pahan K. Regulation of cyclic AMP response element binding and hippocampal plasticity-related genes by peroxisome proliferator-activated receptor alpha. *Cell Rep.* 2013;4(4):724-37.

Legends to supplementary figures:

Figure S1. Monitoring endotoxin level in tau preformed fibrils (PFF). A standard curve (A) was made and levels of endotoxin were measured in LPS (positive control) and PFF (25 and 50 nM) (B) using the ToxinSensor™ Endotoxin Detection System kit (Genscript). Results are mean ± SD of three independent experiments. ****p < 0.001 vs LPS*.

Figure S2. Tau preformed fibrils (PFF) induce the production of TNFα and IL-1β in primary mouse microglia via TLR2. Microglia isolated from *wild type (WT)*, *TLR2*$^{-/-}$ and *TLR4*$^{-/-}$ mice were stimulated with different concentrations of Tau PFF for 24 h under serum-free condition followed by measuring the levels of TNFα (A) and IL-1β (B) proteins in supernatant via ELISA. Results are mean ± SD of three independent experiments. ****p < 0.001 vs respective control*.

Figure S3. Tau preformed fibrils (PFF) activate astrocytes via TLR2. Astrocytes isolated from wild type (*WT*), *TLR2*$^{-/-}$ and *TLR4*$^{-/-}$ mice were stimulated with different concentrations of Tau PFF for 5 h under serum-free condition followed by monitoring the mRNA expression of IL-1β (A), TNFα (B) and iNOS (C) by real-time PCR. Astrocytes plated in 24-well plates at 60-70% confluence were transfected with 0.125 µg pIL-1β promoter-Luc (D), pTNFα promoter-Luc (E) and piNOS promoter-Luc (F), separately using Lipofectamine-Plus. After 24 h of transfection, cells were stimulated with different concentrations of Tau PFF for 4 h under serum-free condition followed by assaying luciferase activity in total cell extracts. Results are mean ± SD of three independent experiments. ****p < 0.001 vs control*. Astrocytes were stimulated with different concentrations of Tau PFF for 18 h under serum-free condition followed by double-labeling for GFAP and iNOS (G). Results represent three independent experiments. Mean fluorescence intensity (MFI) of iNOS was measured (H) by NIH Image J in images from three different experiments. ****p < 0.001*.

Figure S4. AD brain-derived tau (AD-Tau) activates primary mouse microglia via TLR2. Microglia isolated from wild type (*WT*) and *TLR2*$^{-/-}$ mice were stimulated with different concentrations of AD-Tau for 18 h under serum-free condition followed by double-labeling for Iba1 and iNOS (A, *WT* microglia; B, *TLR2*$^{-/-}$ microglia). Mean fluorescence intensity (MFI) of Iba1 (C) and iNOS (D) was measured by NIH Image J in two images of each of three trials per group. ****p < 0.001 vs respective control*. Cells were stimulated with different concentrations of AD-Tau for 5 h under serum-free condition followed by monitoring the mRNA expression of iNOS (E) , TNFα (F), IL-1β (G), and CD11b (H) by real-time PCR. Results are mean ± SD of three independent experiments. ***p < 0.01; ***p < 0.001; NS, not significant vs respective control*.

Figure S5. The wtTIDM peptide inhibits AD-Tau-induced activation of primary mouse microglia. Mouse (WT) primary microglia pre-incubated with wtTIDM or mTIDM (5 µM) for 1 h were stimulated with AD-Tau (2 µg/ml) under serum-free condition for 5 h followed by monitoring the mRNA expression of TNFα (A), IL-1β (B) and iNOS (C) by real time PCR. Results are mean ± SD of three independent experiments. After 18 h of stimulation, cells were double-labeled with antibodies against Iba1 and iNOS (D). Mean fluorescence intensity (MFI) of Iba1 (E) and iNOS (F) was measured by NIH Image J in two images of each of three trials per group. ****p < 0.001*.

Figure S6. Expression of TLR2 and MyD88 is increased in microglia of *PS19* mouse brains. Microglia specific expression of TLR2 and MyD88 in hippocampus of 8.5-months-old *PS19* brains was monitored by conducting double immunofluorescence analysis using either anti-TLR2 or anti-MyD88 antibodies along with anti-Iba1 antibodies (A, B). Images are shown at 20X magnification and then zoomed to visualize the localization of the target proteins. The mean fluorescence intensity (MFI) of TLR2 and MyD88 expression was measured using ImageJ and compared to the values obtained from *nTg* brains (C, D). Microgliosis was assessed by counting total number of Iba1+ microglia in the hippocampus of each mouse brain (E). Two sections from each mouse brain were considered for the MFI and counting analyses and the individual values obtained from each section of a mouse brain are shown in the diagram. Unpaired two-tailed t-test was conducted for determining statistical significance. ** and *** indicate $p < 0.01$ and $p < 0.001$ respectively compared to the *nTg* (n=4 animals per group).

Figure S7. Expression of TLR2 and MyD88 is increased in astrocytes of *PS19* mouse brains. Expression of TLR2 and MyD88 in astrocytes of *PS19* (8.5-months-old) mice was measured by double-label immunofluorescence analyses of each of these proteins in GFAP+ astrocytes present in the hippocampus (A, C). Images are shown at 20X magnification. The MFI analyses of TLR2 and MyD88 was carried out using ImageJ and the mean values were compared with that of the *nTg* brains (B, D). Number of astrocytes in the hippocampus of *nTg* and *PS19* mice was assessed by counting GFAP+ cells and presented as number per mm^2 of tissue (E). Two sections from each mouse brain were considered for the MFI and counting analyses and the individual values obtained from each section of a mouse brain are shown in the diagram. Unpaired two-tailed t-test was conducted for determining statistical significance. * and ** indicate $p < 0.05$ and $p < 0.01$ respectively compared to the *nTg* (n=4 animals per group).

Figure S8. Nasal wtTIDM treatment interrupts the interaction between TLR2 and MyD88 *in vivo* in the hippocampus of *PS19* mice. *PS19* mice (7-months-old) were given intranasal administration of wtTIDM or mTIDM (0.1 mg/kg/d) for 1.5 month. Then colocalization of TLR2 and MyD88 in Iba1 +ve microglia in the hippocampus of these mice was monitored by immunofluorescence analysis followed by imaging under super-resolution (airyscan) confocal microscopy under 40X magnification and further zoomed to visualize the association between TLR2 and MyD88 in the microglia (shown with arrows) (A). The MFI of MyD88 (green) present in the vicinity of TLR2 (red) was measured using Fiji. Two sections from each brain was considered for the analysis and the individual values obtained from each section of a mouse brain are shown in the diagram (B). One-way ANOVA followed by Tukey's multiple comparison analysis was performed to determine statistical significance. *$p < 0.05$; ns, not significant; n=4 animals per group.

Figure S9. Nasal wtTIDM inhibits astroglial inflammation in *PS19* mouse brains. *PS19* mice (7-months-old) were given intranasal administration of wtTIDM or mTIDM (0.1 mg/kg/d) for 1.5 month and then activation of astroglial inflammation in hippocampus was monitored by double immunofluorescence analysis of iNOS in GFAP+ cells (A). Images are shown at 20X magnification. The MFI value of iNOS expression in astrocytes was calculated from each section and two sections from each brain were considered for the analysis, where the individual values obtained from each section of a mouse brain are shown in the diagram (B). Similarly, number of GFAP+ cells were counted from each section and shown in the bar diagram (C). One-way ANOVA

followed by Tukey's multiple comparison analysis was performed to determine statistical significance. * and ** indicate $p < 0.05$ and $p < 0.01$ respectively compared to the designated groups, ns indicates non-significant (n=4 animals per group).

Figure S10. Nasal wtTIDM reduces Ser396/Ser404 tau accumulation in the hippocampus of *PS19* mouse brains. *PS19* mice (7-months-old) were given intranasal administration of wtTIDM or mTIDM (0.1 mg/kg/d) for 1.5 month and the level of phospho-Ser396/Ser404 Tau level in hippocampal neurons was assessed by immunohistochemistry using PHF1 antibody. Overall hippocampal region are shown at 5X magnification and the CA3 and DG regions are further represented at 20X magnification (A). Relative optical density (OD) of PHF1 staining compared to the *nTg* mice was measured using Fiji where data obtained from each section (two sections/mouse brain) is presented (B). One-way ANOVA followed by Tukey's multiple comparison analysis was performed to determine statistical significance. ***$p < 0.001$ compared to the designated groups, ns indicates non-significant (n=4 animals per group).

Figure S11. Age-dependent detection of phospho-Ser202-Thr205 tau in the hippocampus of *PS19* mice. A) Immunostaining of AT8 was performed in 6-months and 8.5-months-old mice brain sections to show age-dependent accumulation of phospho-Ser202-Thr205 form of Tau in hippocampal neurons. B) Relative OD of AT8 of 8.5-months-old *PS19* brain compared to the 6-months-old *PS19* animals. AT8 intensity was measured using Fiji. Statistical analysis was conducted using two-tailed paired t-test. **$p<0.01$. Data are shown as mean ± SEM of n=4 mice per group.

Figure S12. Nasal wtTIDM reduces phospho-tau burden in the hippocampus of *PS19* mouse brains. *PS19* mice (7-months-old) were given intranasal administration of wtTIDM or mTIDM (0.1 mg/kg/d) for one month and the level of phospho-tau accumulation in hippocampal neurons was assessed by immunohistochemistry using AT8 antibody specific for Ser202-Thr205 residues of tau. Images were taken from CA3 and DG regions of the experimental *PS19* mice brain (A). Relative optical density (OD) of AT8 staining compared to the *nTg* mice was measured using Fiji (B). Two sections from each mouse brain were considered for the MFI and counting analyses and the individual values obtained from each section of a mouse brain are shown in the diagram. One-way ANOVA followed by Tukey's multiple comparison analysis was performed to determine statistical significance. * and *** indicate $p < 0.05$ and $p < 0.001$ respectively compared to the designated groups, ns indicates non-significant (n=5 animals per group).

Figure S13. Restoration of PSD95 in the CA3 of *PS19* mouse brain by nasal wtTIDM peptide. *PS19* mice (7-months-old) were given intranasal administration of wtTIDM or mTIDM (0.1 mg/kg/d) for 1.5 month followed by evaluating the level of PSD95 in CA3 by double-label immunofluorescence of PSD95 and MAP2 (A). The MFI of PSD95 was measured using NIH ImageJ in two images of each of four mice per group and the individual values obtained from each section of a mouse brain are shown in the diagram (B). **$p < 0.01$; ***$p < 0.001$; ns, not significant.

Figure S14. Nasal wtTIDM peptide restores synaptophysin in the CA3 of *PS19* mouse brains. *PS19* mice (7-months-old) were given intranasal administration of wtTIDM or mTIDM (0.1 mg/kg/d) for 1.5 month followed by evaluating the level of synaptophysin in CA3 by double-label immunofluorescence of synaptophysin and MAP2 (A). The MFI of synaptophysin was measured

using NIH ImageJ in two images of each of four mice per group and the individual values obtained from each section of a mouse brain are shown in the diagram (B). **$p < 0.01$; ***$p < 0.001$; ns, not significant.

Figure S15. TLR2 deficiency inhibits gliosis in *PS19* mouse brains. Brain sections of age-matched (8.5-months-old) *PS19* and *TLR2* deficient *PS19* ($PS19^{\Delta TLR2}$) mice, treated and untreated with wtTIDM peptide, were immunostained for microglia (Iba1) and astrocytes (GFAP) and the numbers of Iba1 +ve cells and GFAP +ve cells in the hippocampus were counted in each section of experimental groups (A, B). Two sections of each mouse brain were used for the staining and counting analyses and the number of cells/mm^2 of tissue are shown (C, D). Images are shown at lower (5X) and higher magnifications (20X). Statistical analyses were performed using one way ANOVA followed by Tukey's post-hoc test. Data are represented as mean ± SEM (n=4 animals/groups).

Figure S16. The wtNBD peptide inhibits IL-1β-induced upregulation of tau in human neurons. Human neurons pre-incubated with wtNBD or mNBD (5 μM) for 1 h were stimulated with IL-1β (10 ng/ml) in neurobasal media containing B27-AO supplement. After 5 h of treatment, the mRNA expression of *MAPT* (A) was monitored by real-time PCR. Results are mean ± SD of three independent experiments. After 12 h of stimulation, cells were double-labeled with antibodies against tau (EP2456Y) and MAP2 (B). Mean fluorescence intensity (MFI) of Tau (C) was measured by NIH Image J in two images of each of three trials per group. ***$p < 0.001$.

Figure S17. Fibrillar Aβ1-42 induces tau expression in human SH-SY5Y cells via NF-κB. (A) Human SH-SY5Y cells were incubated with different concentrations of fibrillar Aβ1-42 for 18 h followed by monitoring the level of tau by Western blot. Actin was run as a loading control. Tau bands were scanned and values (B, variant 1/Actin; C, variant 2/Actin) presented as relative to control. D) Cells were double-labeled with tau and NeuN. E) MFI of tau was measured by NIH Image J in three images of each of three different experiments. F) After different minutes of stimulation with fibrillar Aβ1-42, DNA-binding activity of NF-κB was monitored in nuclear extracts by EMSA. G) Cells were transfected with PBIIx-Luc for 24 h followed by treatment with different concentrations of fibrillar Aβ1-42 and subjected to luciferase assay. H) Cells were treated with wtNBD or mNBD peptides for 30 mins followed by stimulation with 1 μM fibrillar Aβ1-42 under serum-free conditions. After 5 h, the mRNA expression of tau was measured by real-time PCR. I) Cells were transfected with *pMAPT(WT)-Luc* and *pMAPT(mut)-Luc* for 24 h followed by treatment with fibrillar Aβ1-42 and subjected to luciferase assay after 4 h of stimulation. Results are the mean ± S.D. of three separate experiments (n=3). **$p < 0.01$ & ***$p < 0.001$ *vs control*. NS, not significant.

Figure S18. Etiological reagents of neurodegeneration activate tau promoter in human SH-SY5Y cells via NF-κB. Cells were transfected with *pMAPT(WT)-Luc* and *pMAPT(mut)-Luc* for 24 h followed by treatment with different stimuli including MPP^+ (A), HIV-1 Tat (B), HIV-1 gp120 (C), TNFα (D), poly IC (E), flagellin (F), IFN-γ (G), and tau PFF (H) under serum-free condition. After 4 h, firefly luciferase activity was measured in total cell extracts. Results are represented as mean ± S.D. of three separate experiments (n=3). **$p < 0.01$ & ***$p < 0.001$ *vs control*. NS, not significant.

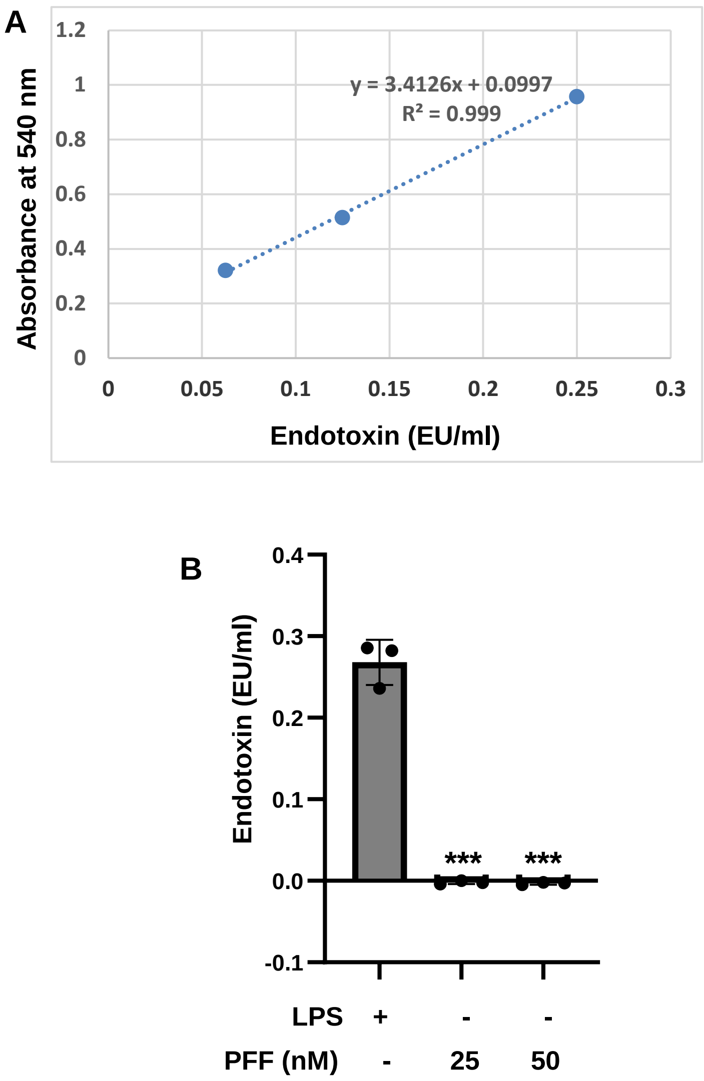
A
1.2
1
0.8
0.6
0.4
0.2
0
Absorbance at 540 nm
y = 3.4126x + 0.0997
R² = 0.999
0
0.05
0.1
0.15
0.2
0.25
0.3
Endotoxin (EU/ml)
B
0.4
0.3
0.2
0.1
0.0
-0.1
Endotoxin (EU/ml)
***
***
LPS + - -
PFF (nM) - 25 50

Figure S1

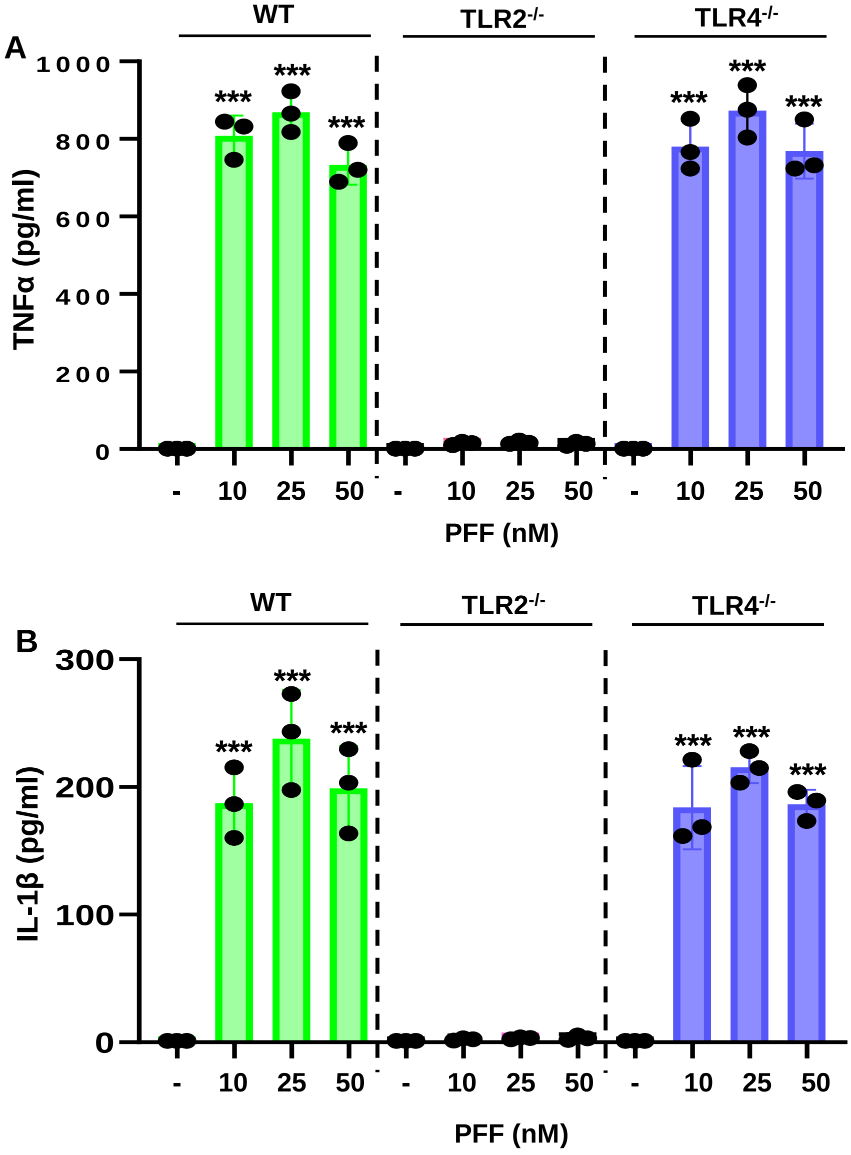

A
WT
TLR2-/-
TLR4-/-
TNFα (pg/ml)
1000
800
600
400
200
0
***
- 10 25 50 - 10 25 50 - 10 25 50
PFF (nM)
B
WT
TLR2-/-
TLR4-/-
IL-1β (pg/ml)
300
200
100
0
***
- 10 25 50 - 10 25 50 - 10 25 50
PFF (nM)

Figure S2

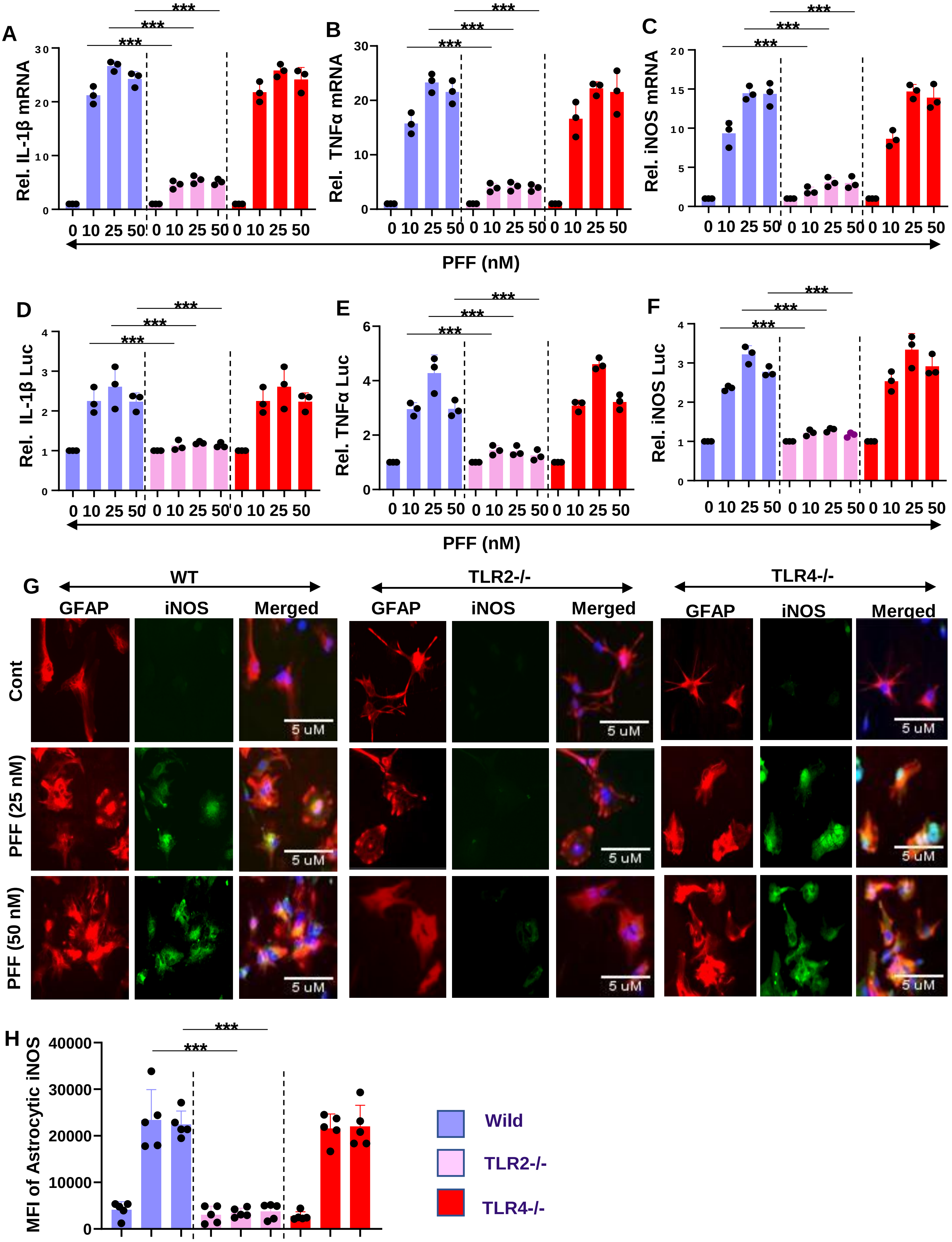
A
B
C
Rel. IL-1β mRNA
Rel. TNFα mRNA
Rel. iNOS mRNA
***
0 10 25 50 0 10 25 50 0 10 25 50
PFF (nM)
D
E
F
Rel. IL-1β Luc
Rel. TNFα Luc
Rel. iNOS Luc
G
WT
TLR2-/-
TLR4-/-
GFAP
iNOS
Merged
Cont
PFF (25 nM)
PFF (50 nM)
5 uM
H
MFI of Astrocytic iNOS
Wild
TLR2-/-
TLR4-/-

Figure S3

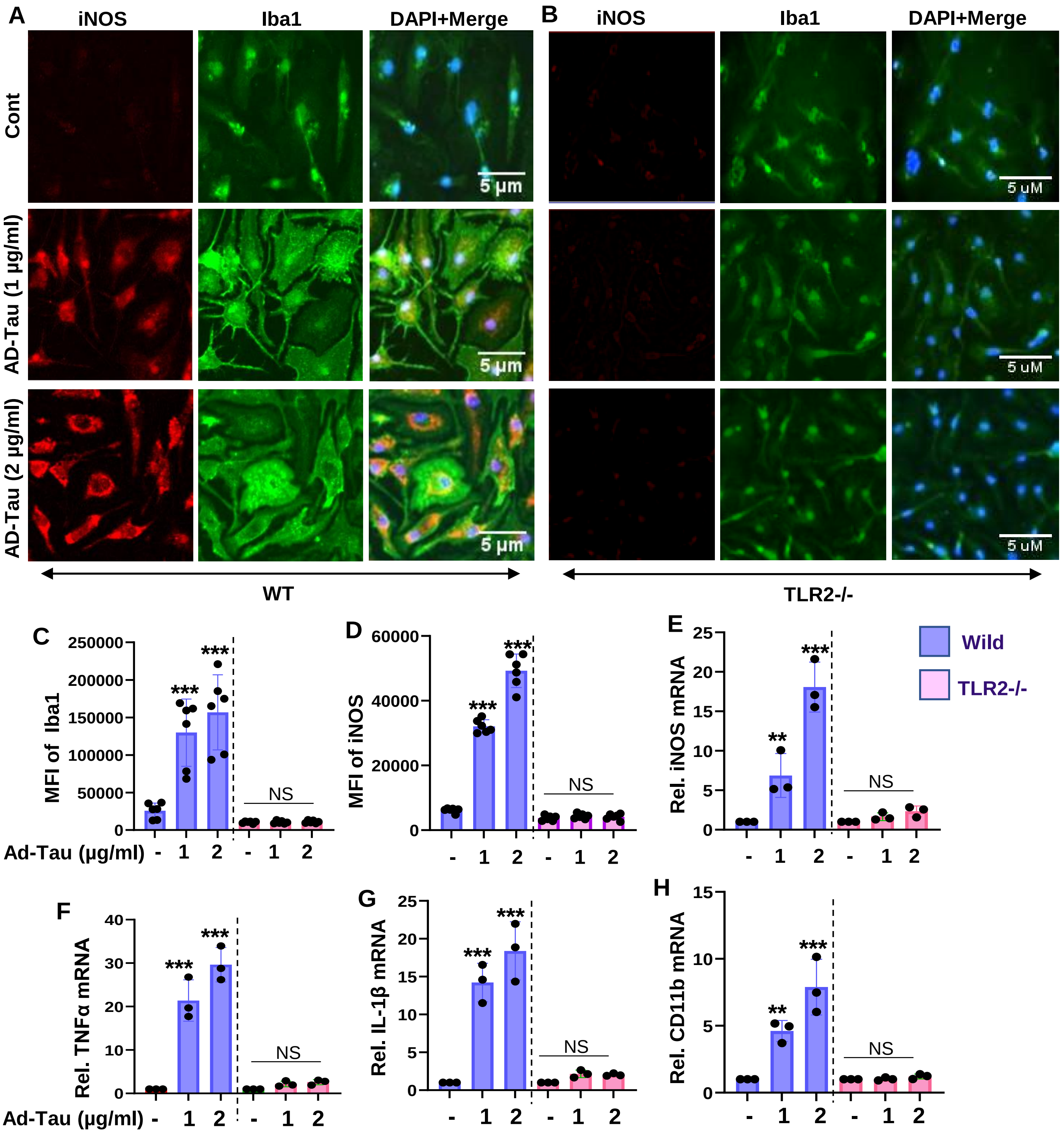

A
iNOS
Iba1
DAPI+Merge
B
iNOS
Iba1
DAPI+Merge
Cont
AD-Tau (1 μg/ml)
AD-Tau (2 μg/ml)
5 μm
5 uM
WT
TLR2-/-
C
MFI of Iba1
250000
200000
150000
100000
50000
0
***
NS
Ad-Tau (μg/ml) - 1 2 - 1 2
D
MFI of iNOS
60000
40000
20000
0
***
NS
- 1 2 - 1 2
E
Rel. iNOS mRNA
25
20
15
10
5
0
**
***
NS
- 1 2 - 1 2
Wild
TLR2-/-
F
Rel. TNFα mRNA
40
30
20
10
0
***
NS
Ad-Tau (μg/ml) - 1 2 - 1 2
G
Rel. IL-1β mRNA
25
20
15
10
5
0
***
NS
- 1 2 - 1 2
H
Rel. CD11b mRNA
15
10
5
0
**
***
NS
- 1 2 - 1 2

Figure S4

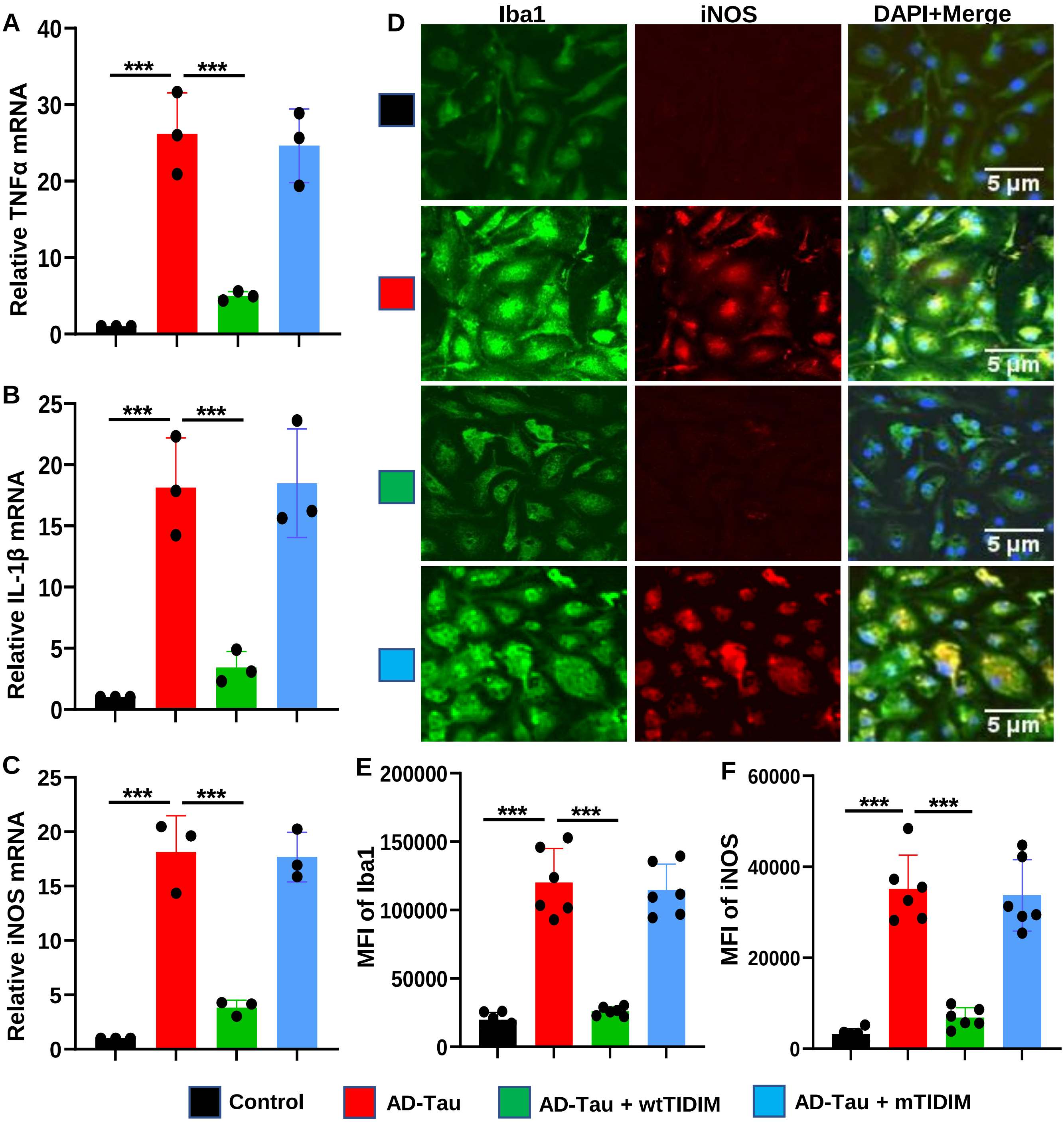
A
B
C
D
E
F
Relative TNFα mRNA
Relative IL-1β mRNA
Relative iNOS mRNA
Iba1
iNOS
DAPI+Merge
5 μm
MFI of Iba1
MFI of iNOS
***
Control
AD-Tau
AD-Tau + wtTIDIM
AD-Tau + mTIDIM

Figure S5

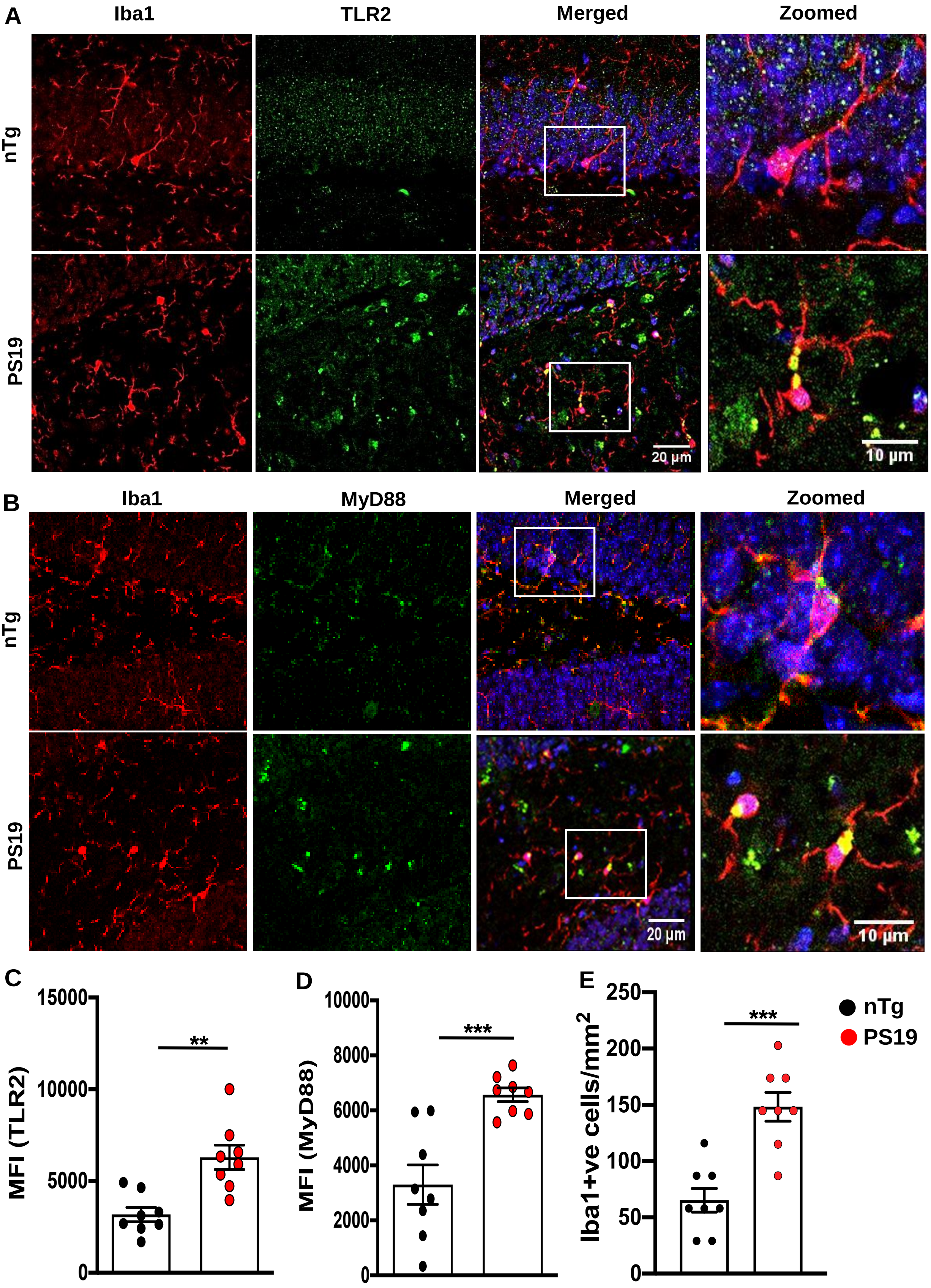

A
Iba1
TLR2
Merged
Zoomed
nTg
PS19
20 µm
10 µm
B
Iba1
MyD88
Merged
Zoomed
nTg
PS19
20 µm
10 µm
C
MFI (TLR2)
15000
10000
5000
0
**
D
MFI (MyD88)
10000
8000
6000
4000
2000
0
***
E
Iba1+ve cells/mm2
250
200
150
100
50
0
***
nTg
PS19

Figure S6

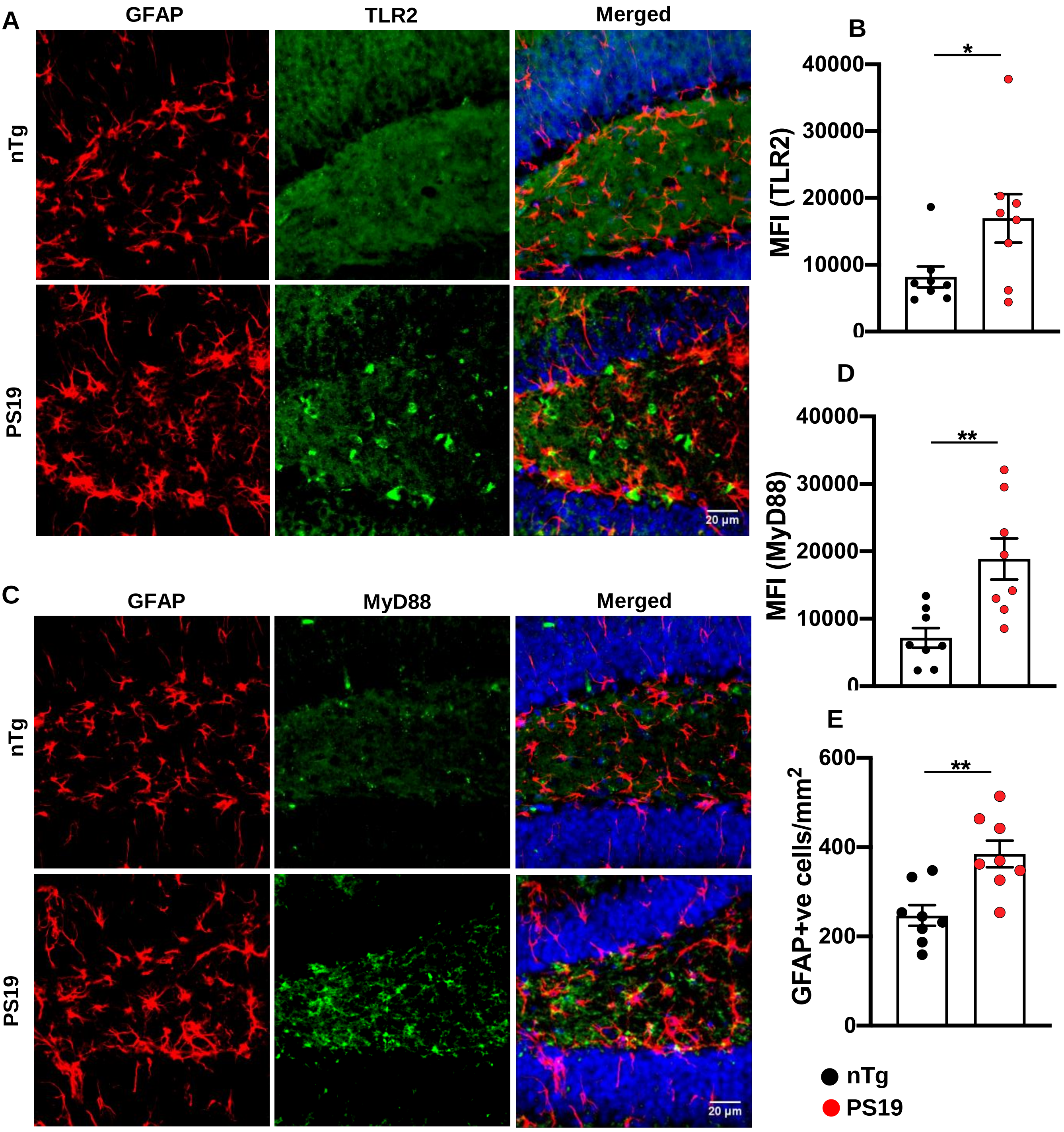
A
GFAP
TLR2
Merged
nTg
PS19
20 μm
B
MFI (TLR2)
40000
30000
20000
10000
0
*
D
MFI (MyD88)
40000
30000
20000
10000
0
**
C
GFAP
MyD88
Merged
nTg
PS19
20 μm
E
GFAP+ve cells/mm2
600
400
200
0
**
nTg
PS19

Figure S7

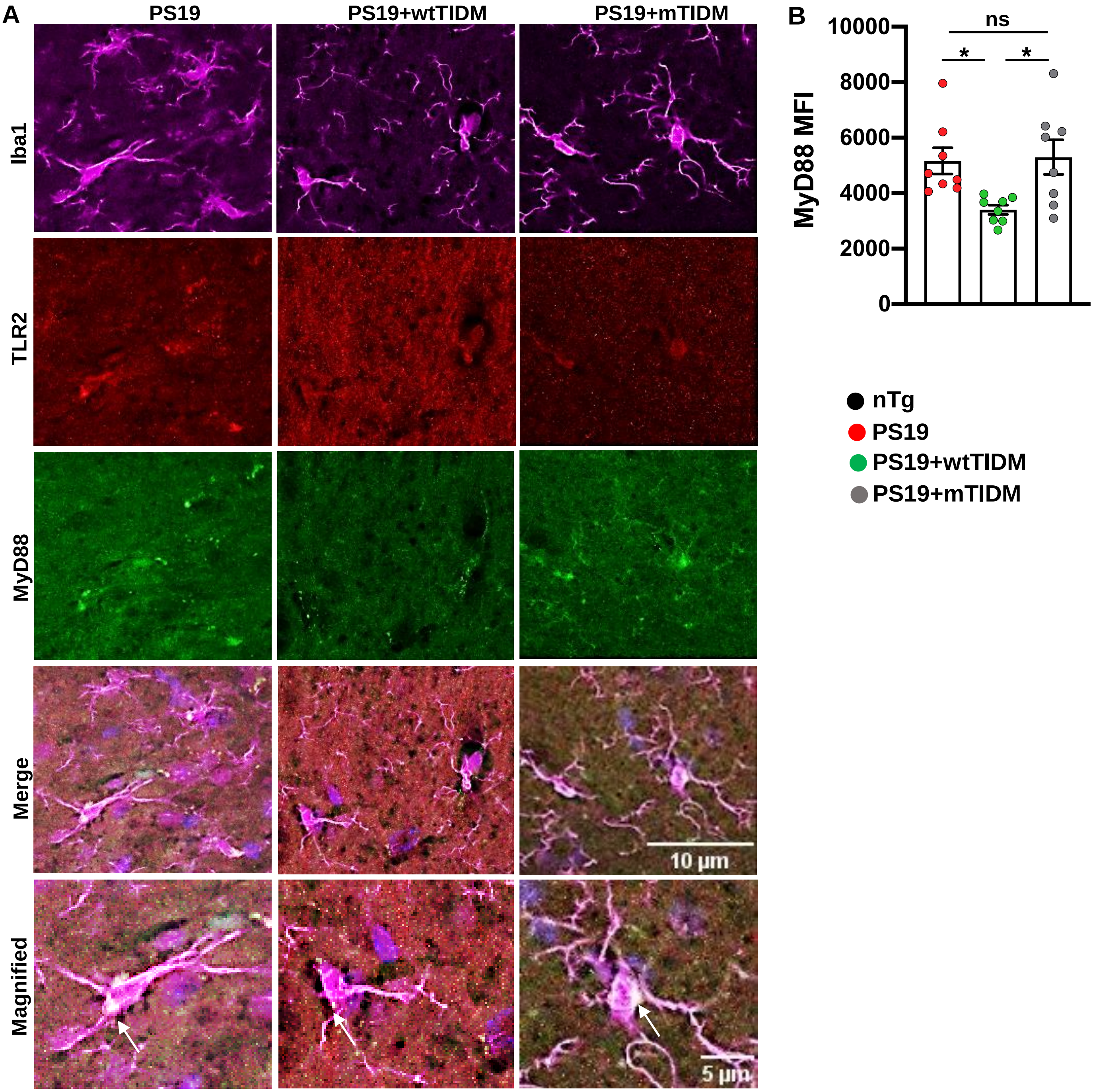
A
PS19
PS19+wtTIDM
PS19+mTIDM
Iba1
TLR2
MyD88
Merge
Magnified
10 µm
5 µm
B
MyD88 MFI
10000
8000
6000
4000
2000
0
ns
*
*
nTg
PS19
PS19+wtTIDM
PS19+mTIDM

Figure S8

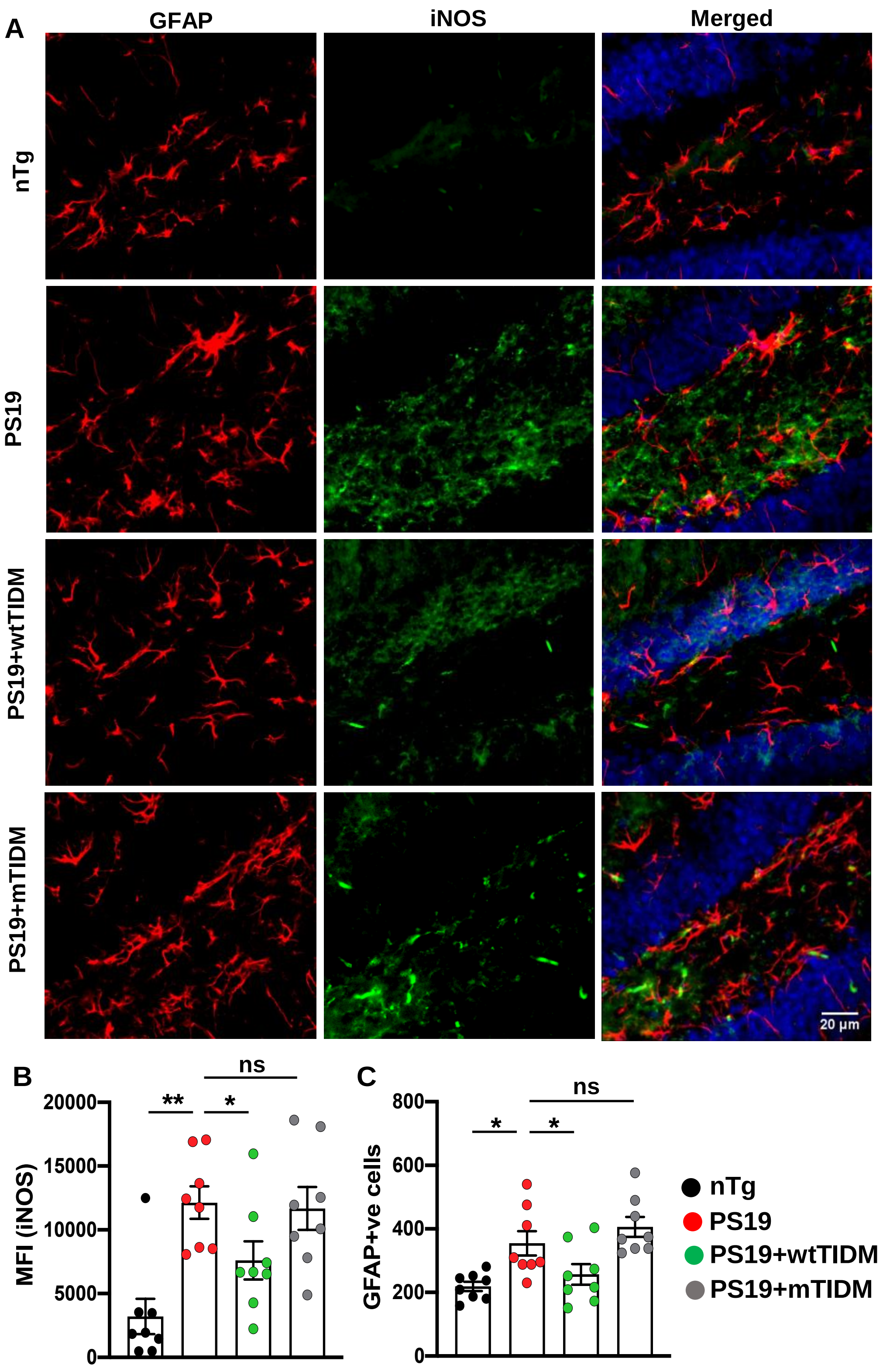
A
GFAP
iNOS
Merged
nTg
PS19
PS19+wtTIDM
PS19+mTIDM
20 µm
B
MFI (iNOS)
20000
15000
10000
5000
0
**
*
ns
C
GFAP+ve cells
800
600
400
200
0
*
*
ns
nTg
PS19
PS19+wtTIDM
PS19+mTIDM

Figure S9

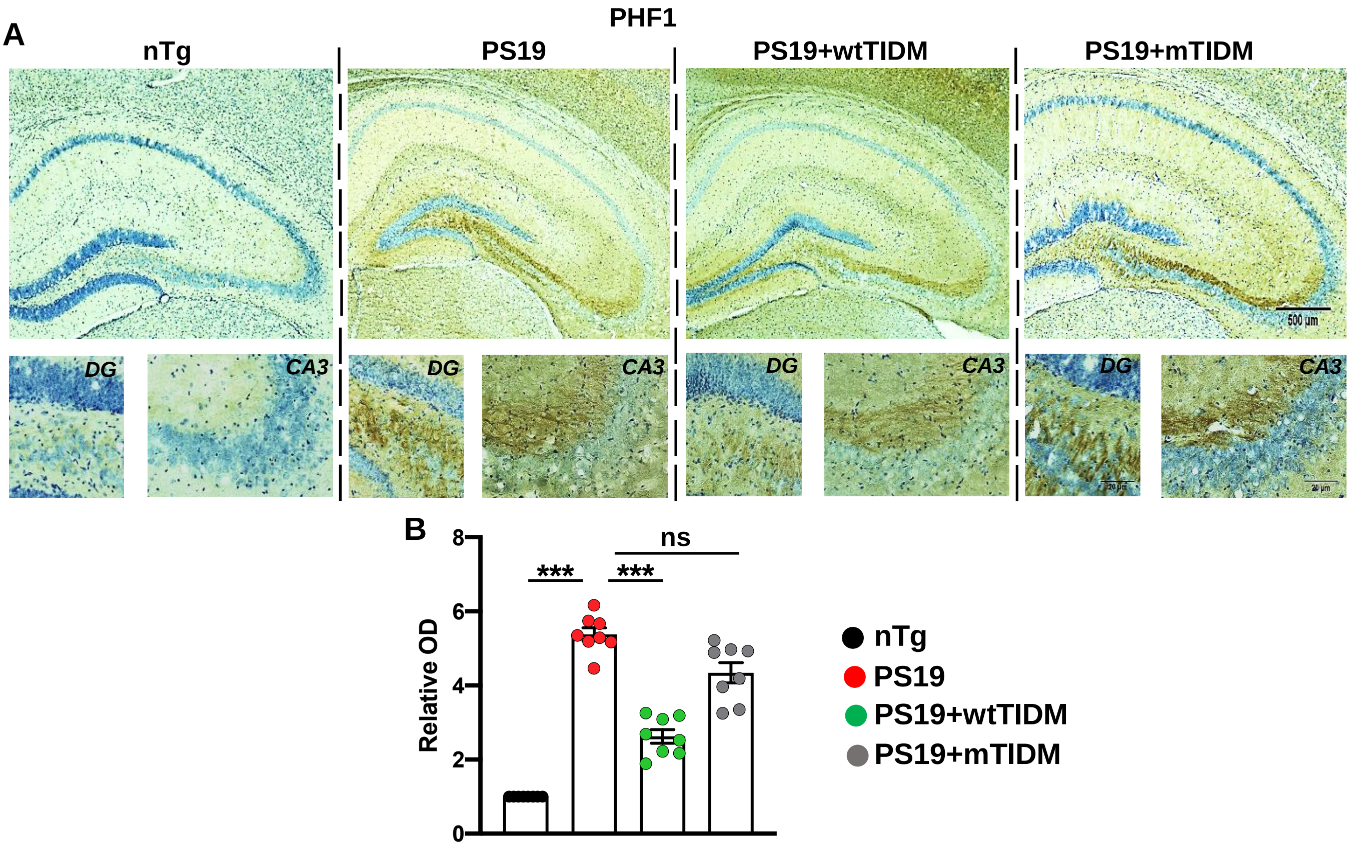
A
PHF1
nTg
PS19
PS19+wtTIDM
PS19+mTIDM
500 μm
DG
CA3
B
Relative OD
8
6
4
2
0
***
***
ns
nTg
PS19
PS19+wtTIDM
PS19+mTIDM

Figure S10

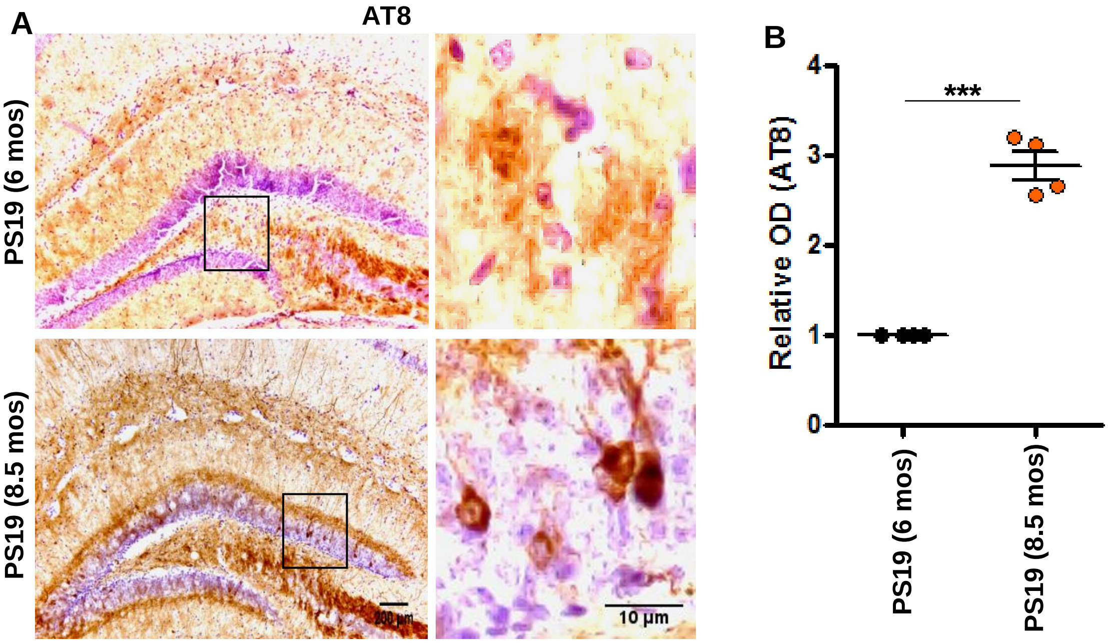
A
AT8
PS19 (6 mos)
PS19 (8.5 mos)
10 µm
B
Relative OD (AT8)
***
0
1
2
3
4
PS19 (6 mos)
PS19 (8.5 mos)

Figure S11

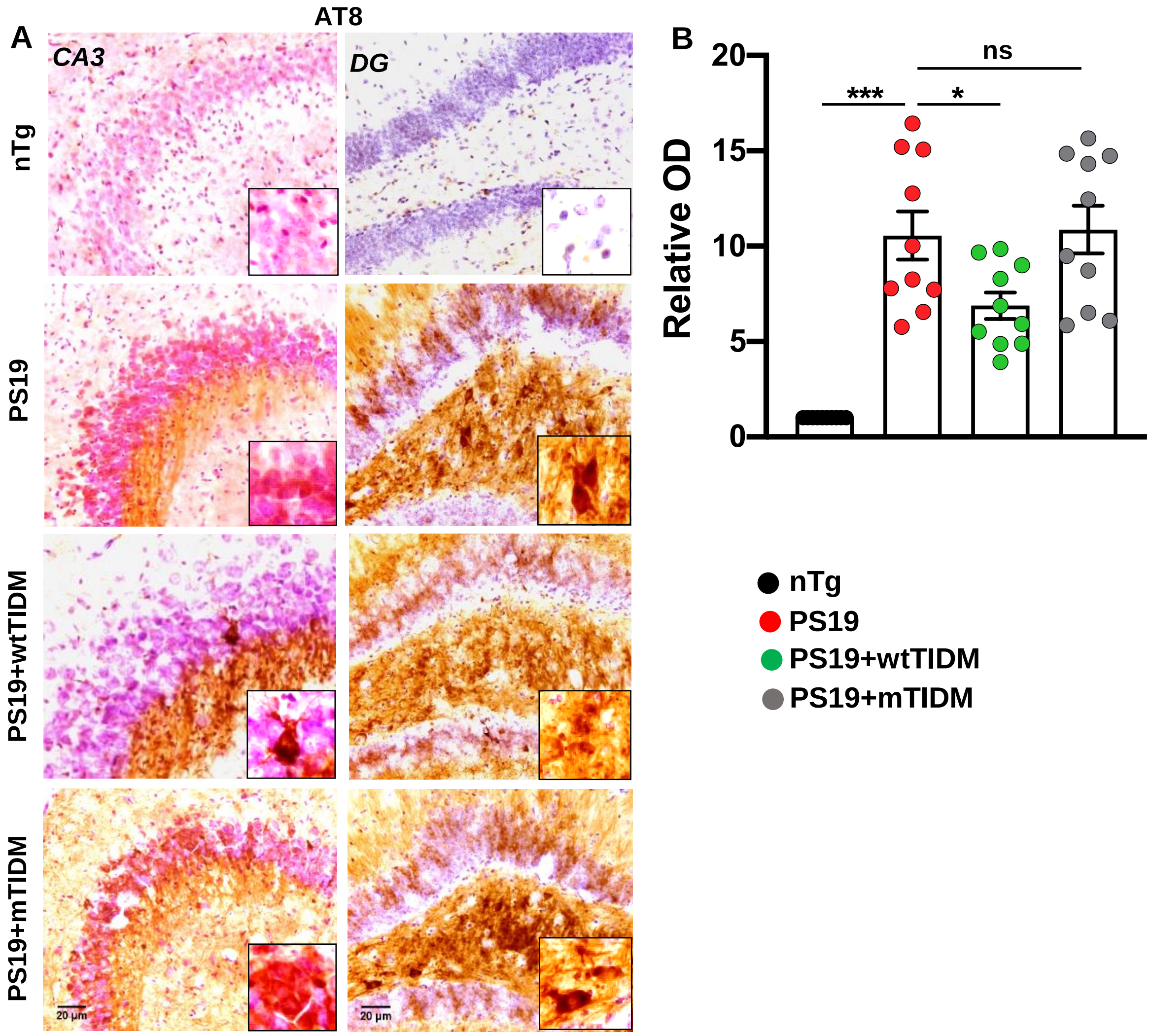

A
AT8
CA3
DG
nTg
PS19
PS19+wtTIDM
PS19+mTIDM
20 µm
20 µm
B
ns
***
*
Relative OD
0
5
10
15
20
nTg
PS19
PS19+wtTIDM
PS19+mTIDM

Figure S12

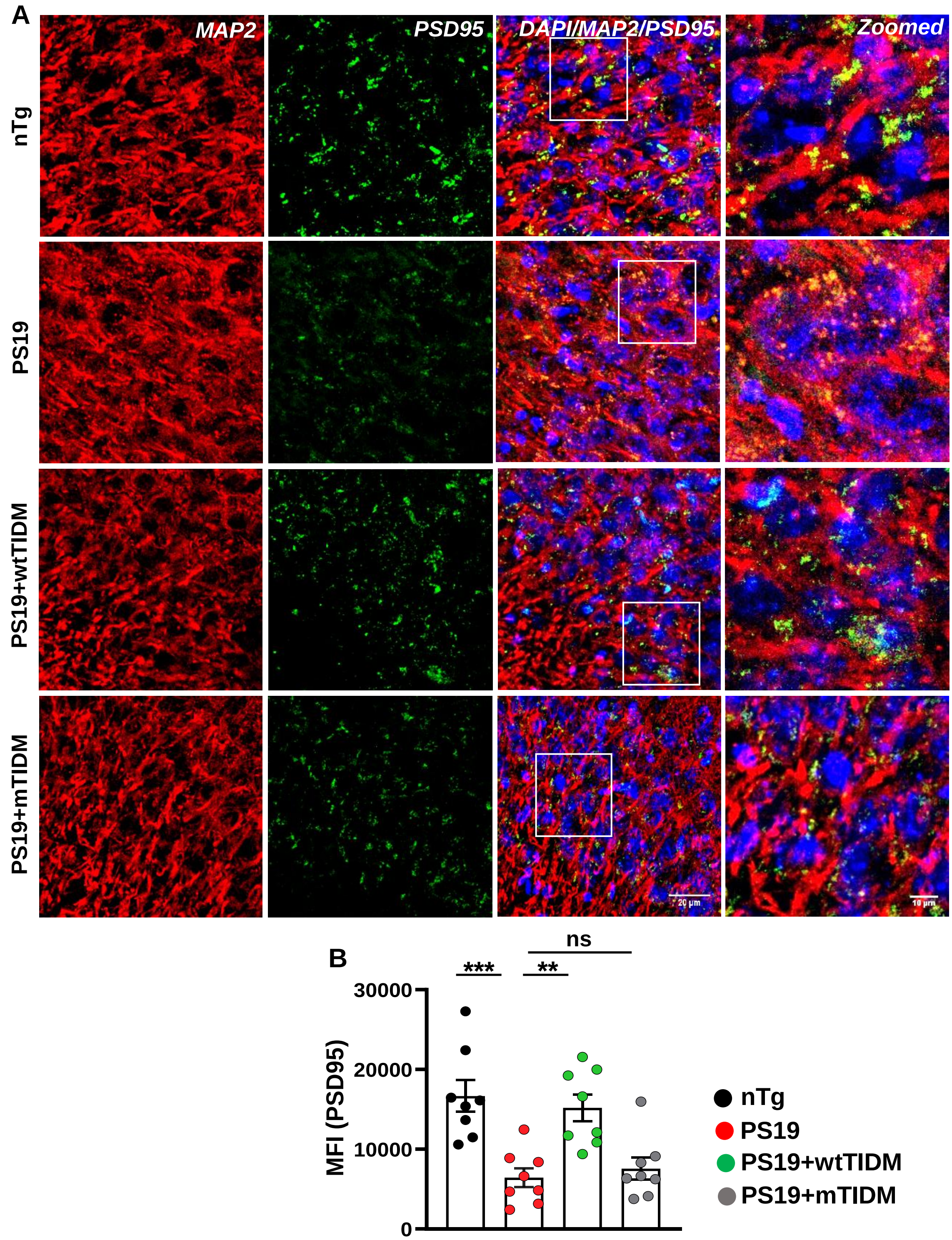
A
MAP2
PSD95
DAPI/MAP2/PSD95
Zoomed
nTg
PS19
PS19+wtTIDM
PS19+mTIDM
B
ns
***
**
30000
20000
10000
0
MFI (PSD95)
nTg
PS19
PS19+wtTIDM
PS19+mTIDM

Figure S13

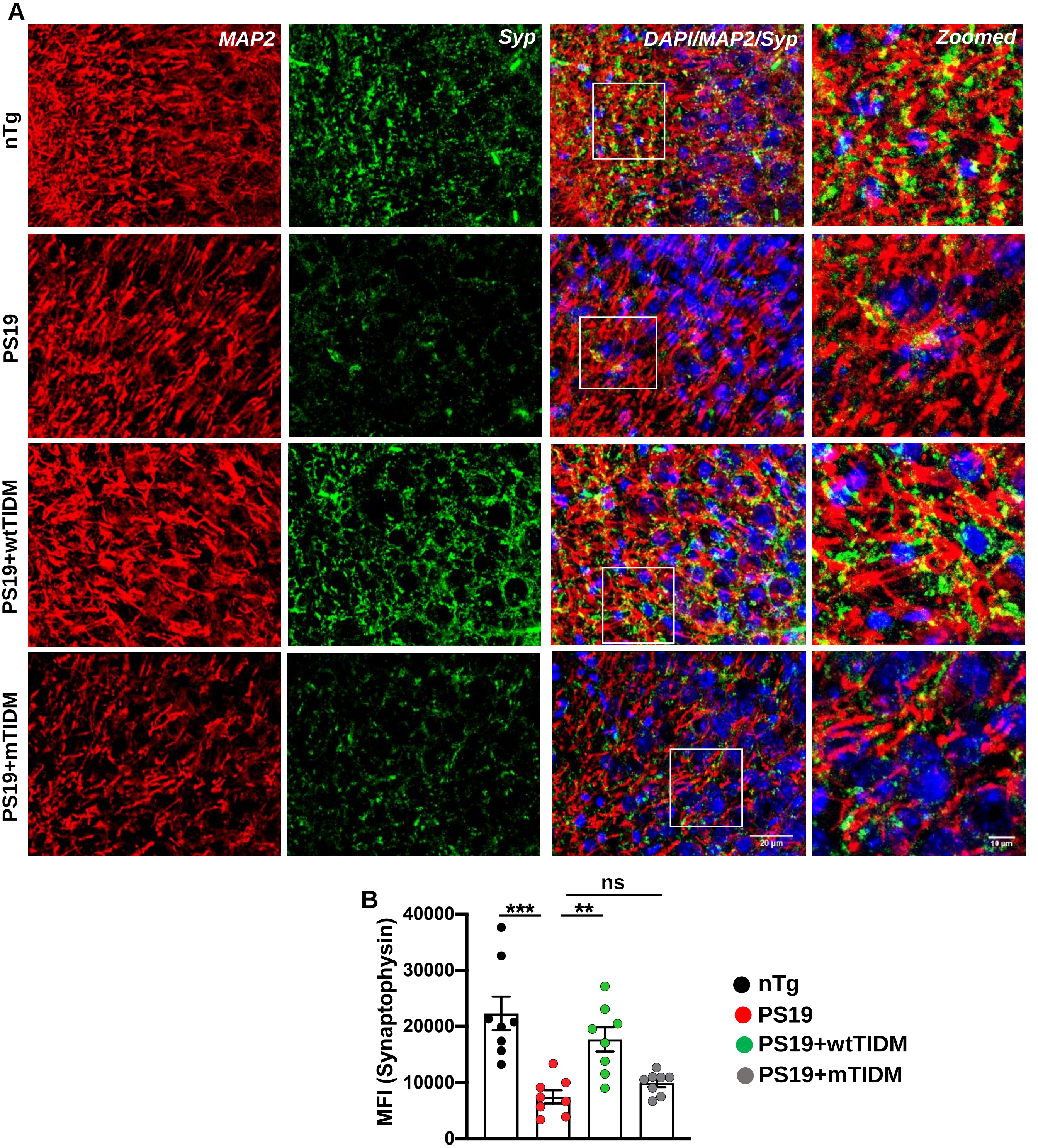
A
MAP2
Syp
DAPI/MAP2/Syp
Zoomed
nTg
PS19
PS19+wtTIDM
PS19+mTIDM
20 μm
10 μm
B
MFI (Synaptophysin)
40000
30000
20000
10000
0
***
**
ns
nTg
PS19
PS19+wtTIDM
PS19+mTIDM

Figure S14

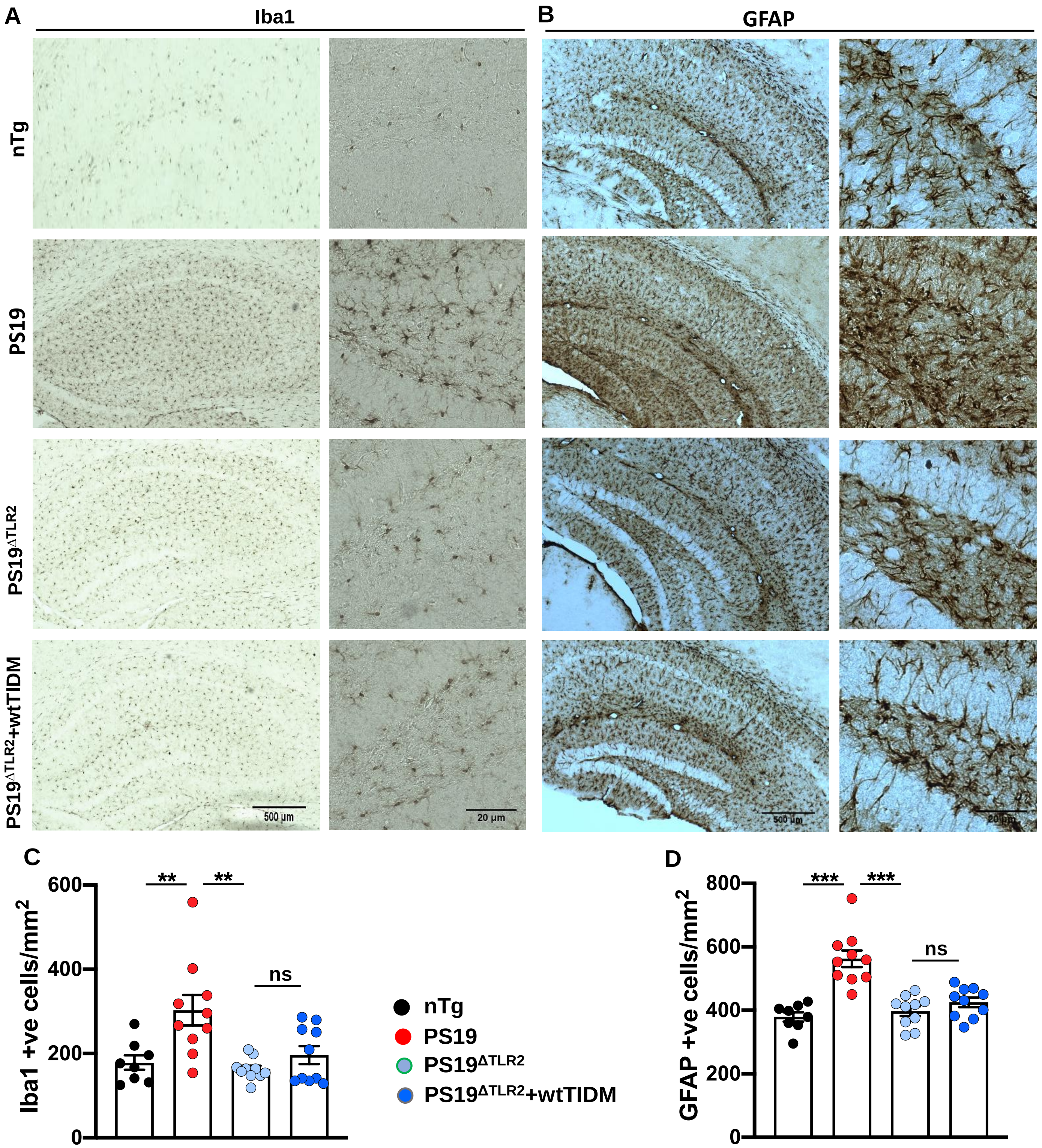
A
Iba1
B
GFAP
nTg
PS19
PS19ΔTLR2
PS19ΔTLR2+wtTIDM
500 µm
20 µm
C
Iba1 +ve cells/mm2
600
400
200
0
**
**
ns
nTg
PS19
PS19ΔTLR2
PS19ΔTLR2+wtTIDM
D
GFAP +ve cells/mm2
800
600
400
200
0
***
***
ns

Figure S15

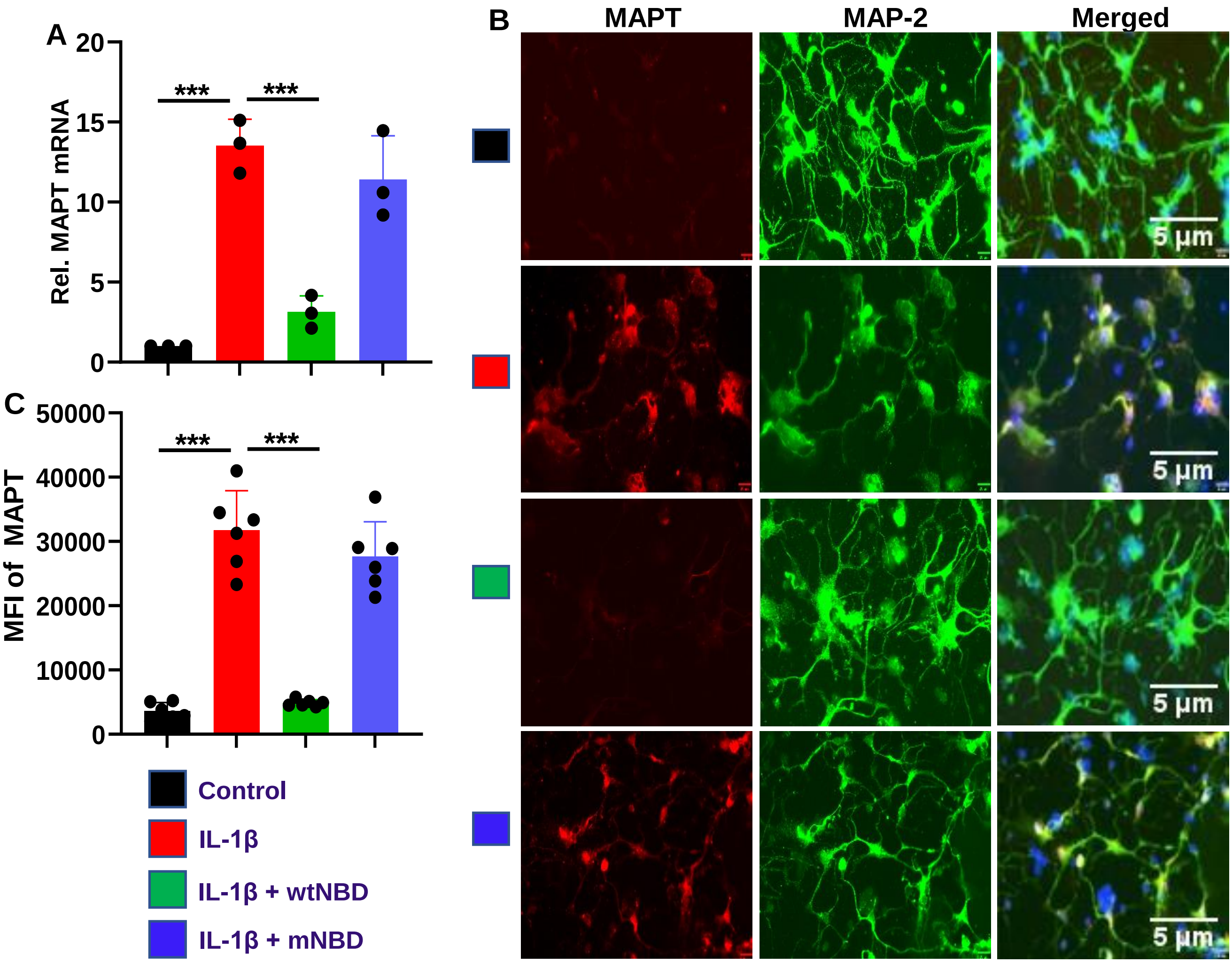
A
B
C
Rel. MAPT mRNA
MFI of MAPT
***
MAPT
MAP-2
Merged
5 µm
Control
IL-1β
IL-1β + wtNBD
IL-1β + mNBD

Figure S16

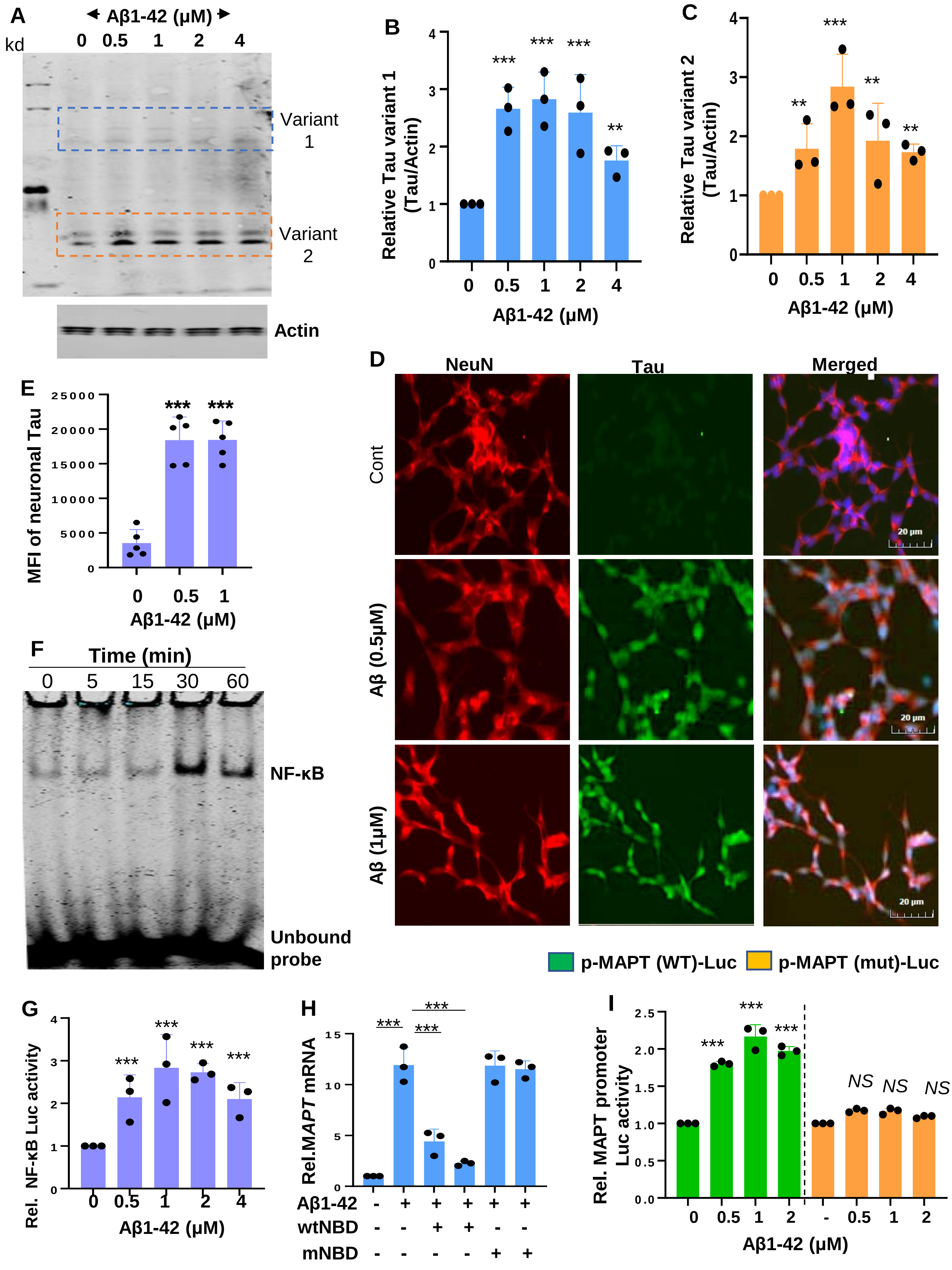
A
Aβ1-42 (μM)
kd
0 0.5 1 2 4
Variant 1
Variant 2
Actin
B
Relative Tau variant 1 (Tau/Actin)
Aβ1-42 (μM)
C
Relative Tau variant 2 (Tau/Actin)
Aβ1-42 (μM)
D
NeuN
Tau
Merged
Cont
Aβ (0.5μM)
Aβ (1μM)
20 μm
E
MFI of neuronal Tau
Aβ1-42 (μM)
F
Time (min)
0 5 15 30 60
NF-κB
Unbound probe
p-MAPT (WT)-Luc
p-MAPT (mut)-Luc
G
Rel. NF-κB Luc activity
Aβ1-42 (μM)
H
Rel.MAPT mRNA
Aβ1-42
wtNBD
mNBD
I
Rel. MAPT promoter Luc activity
NS
Aβ1-42 (μM)

Figure S17

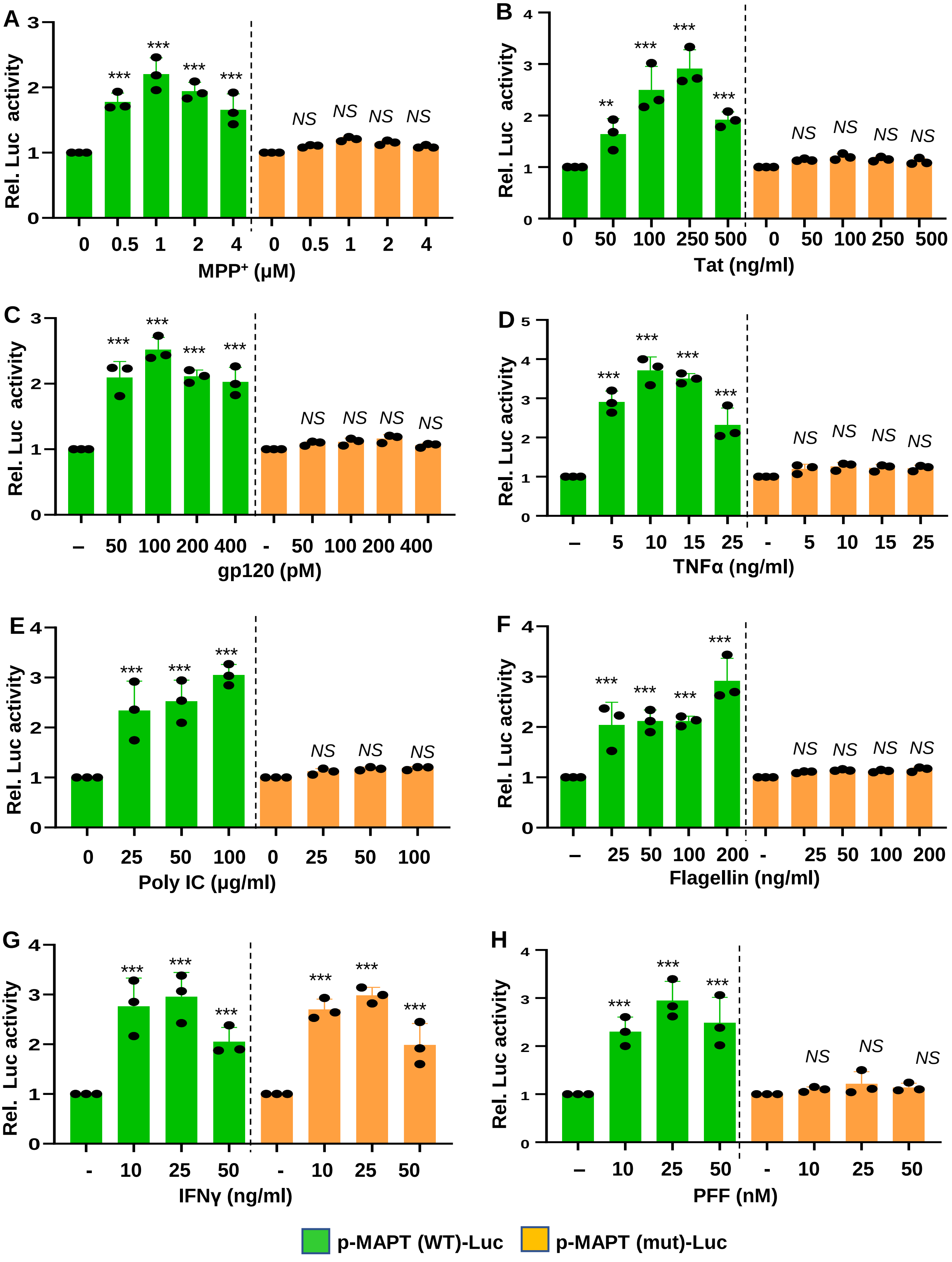
A
Rel. Luc activity
MPP⁺ (μM)
0 0.5 1 2 4 0 0.5 1 2 4
NS NS NS NS
B
Rel. Luc activity
Tat (ng/ml)
0 50 100 250 500 0 50 100 250 500
C
gp120 (pM)
– 50 100 200 400 - 50 100 200 400
D
TNFα (ng/ml)
– 5 10 15 25 - 5 10 15 25
E
Poly IC (μg/ml)
0 25 50 100 0 25 50 100
F
Flagellin (ng/ml)
– 25 50 100 200 - 25 50 100 200
G
IFNγ (ng/ml)
- 10 25 50 - 10 25 50
H
PFF (nM)
– 10 25 50 - 10 25 50
p-MAPT (WT)-Luc
p-MAPT (mut)-Luc

Figure S18

Table S1: Antibodies used for the study.

Protein	Source	Catalogue No.	Application/Dilution	Host Species
Total Tau	Thermo Fischer	AHB0042	WB/1:1000<br>IHC/1:500	Mouse
Phospho-tau	Thermo Fischer	MN1020	IHC/1:750	Mouse
Synthetic tau peptide	Abcam	Ab76128	IFC/1:500	Rabbit
Inducible nitric oxide synthase (iNOS)	BD Bioscience	610329	WB/1:1000<br>IFC/1:200	Mouse
Ionized calcium binding adaptor molecule 1 (Iba1)	Abcam	ab5076	WB/1:1000<br>IFC/1:1000	Goat
Glial fibrillary acidic protein (GFAP)	Abcam	ab53554	IFC/1:1000	Goat
MyD88	Santa Cruz	sc-74532	IFC/1:250<br>IP/	Mouse
Toll like receptor 2 (TLR2)	Abcam	ab16894	WB/1:500<br>IFC/1:250	Mouse
PhosphoSer536 p65	Cell Signaling	3033S	IFC/1:250	Rabbit
TLR4	Abcam	ab13867	WB/1:500	Rabbit
Actin	Abcam	ab8226	WB/1:10000	Mouse

Antibodies used for the study. The Table shows name, source, catalog no. and dilutions of the antibodies. WB, Western blot; IFC, Immunofluorescence, IHC, Immunohistochemistry.
